# A Cascade of Iron-Containing Proteins Governs the Genetic Iron Starvation Response to Promote Iron Uptake and Inhibit Iron Storage in Fission Yeast

**DOI:** 10.1371/journal.pgen.1005106

**Published:** 2015-03-25

**Authors:** Javier Encinar del Dedo, Natalia Gabrielli, Mercè Carmona, José Ayté, Elena Hidalgo

**Affiliations:** Oxidative Stress and Cell Cycle Group, Universitat Pompeu Fabra, Barcelona, Spain; The University of North Carolina at Chapel Hill, United States of America

## Abstract

Iron is an essential cofactor, but it is also toxic at high levels. In *Schizosaccharomyces pombe*, the sensor glutaredoxin Grx4 guides the activity of the repressors Php4 and Fep1 to mediate a complex transcriptional response to iron deprivation: activation of Php4 and inactivation of Fep1 leads to inhibition of iron usage/storage, and to promotion of iron import, respectively. However, the molecular events ruling the activity of this double-branched pathway remained elusive. We show here that Grx4 incorporates a glutathione-containing iron-sulfur cluster, alone or forming a heterodimer with the BolA-like protein Fra2. Our genetic study demonstrates that Grx4-Fra2, but not Fep1 nor Php4, participates not only in iron starvation signaling but also in iron-related aerobic metabolism. Iron-containing Grx4 binds and inactivates the Php4 repressor; upon iron deprivation, the cluster in Grx4 is probably disassembled, the proteins dissociate, and Php4 accumulates at the nucleus and represses iron consumption genes. Fep1 is also an iron-containing protein, and the tightly bound iron is required for transcriptional repression. Our data suggest that the cluster-containing Grx4-Fra2 heterodimer constitutively binds to Fep1, and upon iron deprivation the disassembly of the iron cluster between Grx4 and Fra2 promotes reverse metal transfer from Fep1 to Grx4-Fra2, and de-repression of iron-import genes. Our genetic and biochemical study demonstrates that the glutaredoxin Grx4 independently governs the Php4 and Fep1 repressors through metal transfer. Whereas iron loss from Grx4 seems to be sufficient to release Php4 and allow its nuclear accumulation, total or partial disassembly of the Grx4-Fra2 cluster actively participates in iron-containing Fep1 activation by sequestering its iron and decreasing its interaction with promoters.

## Author Summary

Iron is an essential biometal but it is also toxic, and therefore its intracellular availability from disposable iron pools is tightly regulated. From bacteria to higher eukaryotes, iron starvation triggers complex genetic responses to exacerbate the otherwise limited iron uptake and decrease intracellular iron storage and usage. These responses are triggered in very distinct ways in each organism. In fission yeast, two transcriptional repressors, Php4 and Fep1, mediate the iron usage/iron import cellular response to iron starvation, respectively, and a glutaredoxin Grx4-Fra2 heterodimer governs both repressors. We show here that iron is an essential component of the Grx4-Fra2 heterodimers and of the transcriptional repressor Fep1. Under normal iron conditions, iron-containing Grx4 maintains Php4 retained in the cytosol, and iron depletion forces their dissociation and Php4 nuclear accumulation. On the other hand, iron-bridged Grx4-Fra2 is bound to Fep1 at repressed promoters, and iron depletion forces reverse metal transfer from Fep1 to Grx4-Fra2, and transcriptional de-repression. These complex molecular events occur upon iron scarcity to induce iron import and decrease iron usage, and explains how a single protein complex, Grx4-Fra2, can both activate and inactivate transcription to mount a survival response.

## Introduction

Since iron (Fe) is essential but also toxic, its uptake from the extracellular environment and its intracellular availability from a “disposable Fe pool” are tightly regulated. All cell types display wide transcriptome changes upon Fe starvation. These responses are triggered in very distinct ways in each organism, but the final gene expression programs are quite similar in essence: they are meant to temporally increase Fe import and decrease Fe storage and usage.

**Fig 1:**
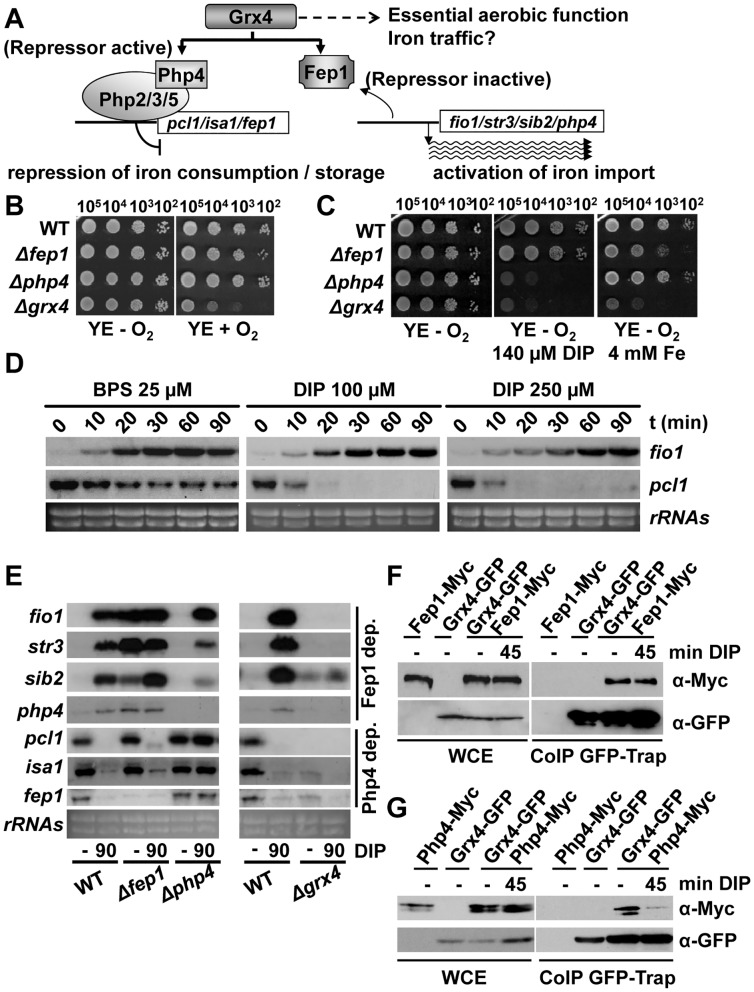
The glutaredoxin Grx4 functions in both Fe signaling and Fe traffic. (A) Scheme of the putative roles of Grx4 in Fe delivery and Fe signaling. Grx4 as a Fe sensor regulates the repressors Fep1 and Php4. (B) Only cells lacking Grx4 display growth defects under aerobic conditions. Strains 972 (WT), NG2 (*Δfep1*), NG40 (*Δphp4*) and NG81 (*Δgrx4*) were spotted and grown on YE plates under aerobic or anaerobic conditions. (C) Cells lacking Fep1, Php4 or Grx4 display growth defects in the presence of Fe chelators or Fe excess. Strains as in B were spotted and grown under anaerobic conditions on plates containing or not DIP or ammonium ferrous sulfate (Fe). (D) The Fe chelators BPS and DIP trigger the activation of the Fe starvation response with different kinetics. Total RNA was obtained from YE cultures of wild-type strain 972 treated or not with 25 μM BPS, 0.1 mM DIP or 0.25 mM DIP for the indicated times in minutes, and analyzed by Northern blot with the probes indicated. *rRNA* was used as a loading control. (E) Php4, Fep1 and Grx4 are essential for the induction of the Fe starvation response. Total RNA from strains as in B was obtained from YE cultures treated or not with 0.25 mM DIP for 90 min, and analyzed by Northern blot with the probes indicated. *rRNA* was used as a loading control. (F and G) The interaction of Grx4 with Php4, but not with Fep1, is disturbed upon Fe starvation. (F) Strains NG108 (*fep1-myc*), NG115 (*grx4-GFP*) and NG109 (*grx4-GFP fep1-myc*) were treated or not with 0.25 mM DIP for the indicated times. Total native protein extracts were immuno-precipitated with GFP-trap beads. Immuno-precipitates were analyzed by SDS–PAGE and blotted with anti-Myc or anti-GFP antibodies. As a loading control, whole-cell extracts were loaded (WCE). (G) Strains NG107 (*php4-myc*), NG115 (*grx4-GFP*) and NG120 (*grx4-GFP php4-myc*) were treated as described in F.

In *Schizosaccharomyces pombe*, the repressors Fep1 and Php4 mediate the transcriptional response to Fe depletion [1] (Fig. 1A). When Fe is not limiting, Fep1 represses the expression of several genes which mediate Fe uptake and/or increase the intracellular available Fe pool, such as those coding for the reductive high-affinity transporter Fio1 [2], the non-reductive importer Str3[3] or the ferrichrome sinthetase Sib2 [4]. Fep1 de-represses transcription of these genes under Fe deprivation [5], but is localization remains nuclear [6]. Php4, on the contrary, has Crm1-dependent cytosolic localization under basal conditions. When Fe is scarce, Php4 accumulates in the nucleus and represses transcription of genes activated by the Pho2/3/5 complex, acting as a transcriptional repressor [7]. These more than 80 repressed genes, according to microarray analysis [8], include those coding for the vacuole Fe importer Pcl1, the Fe-sulfur (Fe-S) cluster-containing protein Sdh4 and the Fe-S cluster assembly protein Isa1 [1]. Fep1 also represses the *php4* gene under basal conditions [1], whereas Php4 blocks *fep1* expression under Fe depleted conditions [8]. In *Saccharomyces cerevisiae*, the Fe starvation response is based on the activation of the positive transcription factors Aft1/2, and in post-transcriptional regulation of mRNA stability by the RNA-binding proteins Cth1/2 (for a recent review, see [9]). The only common element in the cascades of budding and fission yeast seems to be the Fe sensor glutaredoxin 4 (Fig. 1A).

The *S*. *cerevisiae* redundant glutaredoxins Grx3/4 have been described to participate not only in Fe sensing, but also in metal delivery to Fe-containing proteins [10]. Grx3/4 are monothiol glutaredoxins containing an Fe-S cluster between two subunits of the glutaredoxins or between a heterodimer with the bacterial BolA-like protein Fra2 (for a review, see [11]). Regarding Fe signaling, Fe deprivation promotes nuclear accumulation of a positive transcription factor, Aft1/2 [12,13], after disassembly from the apo-Grx4-Fra2 heterodimer [14,15]. In fission yeast, there is only one cytosolic monothiol glutaredoxin, Grx4, which was first described to be essential for growth [16], although cells devoid of Grx4 can grow under semi-anaerobic conditions[7]. Grx4 is a repressor of Php4 activity under basal conditions, since deletion of *grx4* triggers constitutive nuclear localization of Php4 and repression of genes such as *pcl1* under Fe-rich conditions [7]. Fep1 is also regulated by Grx4: in the absence of this glutaredoxin, the expression of Fep1-dependent genes such as *fio1* cannot be induced [17,18]. Grx4 contains an N-terminal thioredoxin domain and a C-terminal glutaredoxin domain, each one of which containing a unique cysteine (Cys) residue. The role of their thiol groups in the protein’s function as an Fe sensor has been studied with conditional mutants or tagged versions of Grx4 with conflicting results: the interaction with Fep1 is disturbed in one of the Cys mutants according to one of the reports [18] but not the other [17].

The molecular events connecting Fe levels and activity of this signaling cascade are unknown. We have performed a biochemical and genetic study of the different components of the pathway. Not only Grx4 but also Fep1 are Fe-binding proteins. We have purified recombinant proteins and show here that there is a Fe-S bridging cluster between two Grx4 subunits and between Grx4 and Fra2, a new component of this *S*. *pombe* cascade. Genetic data suggest that Fe-containing Grx4 homodimer is sufficient for cytosolic retention of Php4, although Fra2 seems to partially contribute to this retention; Fe starvation should promote accumulation of apo-Grx4, and release of Php4. On the contrary, the Fe-S cluster bridging Grx4 and Fra2 is required for regulation of Fep1 activity; upon Fe starvation, total or partial disassembly of this cluster may sequester Fe from Fep1 and decrease its affinity for DNA.

## Results

### Fission yeast Grx4 is required for sensing Fe starvation

In *S*. *cerevisiae*, the redundant proteins Grx3/4 regulate two important processes of cell survival and adaptation: delivery of Fe to proteins and regulation of the transcription factor Aft1, activator of a transcriptional response to Fe starvation [12,13] (for a review, see [11]). To confirm whether the same dual function applies to the *S*. *pombe* homolog Grx4 (Fig. 1A), we generated a *Δgrx4* strain by selection under anaerobic conditions, and tested first the effect of such gene deletion in growth. As shown in Fig. 1B, the lack of Grx4 jeopardizes the cell’s capacity to grow on plates in the presence of oxygen even in rich media (YE), and especially in the respiratory-prone minimal media (MM). Php4 and Fep1 are dispensable for this aerobic function (Fig. 1B). Similarly, liquid aerobic growth is impaired only in cells lacking Grx4.

Regarding Fe signaling, we first compared the effect of different Fe chelators in the growth of fission yeast. We tested chelators such as the membrane-permeable dipyridyl (DIP), desferroxamine (Dx), a siderophore which chelates Fe extracellularly, or the extracellular Fe chelator bathophenanthroline disulfonate (BPS). As shown in Supplemental information (S1A Fig), addition of different concentrations of these chelators impairs or fully halts the growth of wild-type *S*. *pombe* cultures. DIP is the only compound able to immediately cease the growth on fission yeast in liquid cultures, while the extracellular chelators allowed several cell cycles since they only blocked iron acquisition by eliminating the extracellular iron sources.

Using as a reference the sub-toxic concentrations on liquid cultures for the different chelators (that is, those that reduced the growth rates but did not fully inhibit growth), we then tested on solid plates that cells lacking Php4 or Grx4, but not *Δfep1* cells, were more sensitive than wild-type strain to grow in the presence of DIP (Fig. 1C), Dx or BPS (S1B Fig). On the contrary, an excess of Fe only affects cells lacking Fep1 or Grx4 (Fig. 1C).

To explain these phenotypes, we first analyzed the response of wild-type cells to two different chelators, BPS and DIP. While activation of Fep1-dependent genes such as *fio1* occurred with similar kinetics upon treatment with BPS or DIP at low and high concentrations, repression of Php4-genes such as *pcl1* was much weaker in the presence of the extracellular chelator BPS than with the permeable DIP (Fig. 1D): BPS treatment induced repression of *pcl1* at lower rates and its mRNA never reached the levels accomplished by DIP treatment.

In order to test whether Grx4 is also essential for the induction of the Fep1- and Php4-dependent changes of gene expression upon Fe deprivation, we decided to use high doses of DIP (250 μM) to highlight the effects on Php4-dependent genes, which are more dramatic with this chelator. Furthermore, most work in the characterization of the iron starvation response in *S*. *pombe* has been performed using DIP at this concentration (see [1,8], and references there-in). As shown in Fig. 1E, in the presence of DIP wild-type cells up-regulate transcription of Fep1-dependent genes, whereas they down-regulate the expression of Php4-dependent ones. The inactivation of the Fep1 and Php4 repressors upon DIP and basal conditions, respectively, is dependent on Grx4, since *Δgrx4* cells can not signal-activate genes such as *fio1*, *str3*, *sib2* or *php4* and constitutively repress genes such as *pcl1*, *isa1* or *fep1* (Fig. 1E).

We then confirmed with immuno-fluorescence (S2A Fig) or fluorescent microscopy (S2B Fig) that Grx4 is localized at both the cytosol and the nucleus, Fep1 is constitutively nuclear, and Php4 shifts from the cytosol to the nucleus upon Fe deprivation. Using double tagged strains, we then tested whether Grx4-GFP would interact with the repressors before and/or after stress. We immuno-precipitated Grx4-GFP, and used commercial antibodies against the Myc-tag to check the *in vivo* binding to Php4-Myc (Fig. 1G) or Fep1-Myc (Fig. 1F). As shown in Fig. 1G, the association between Grx4-GFP and Php4-Myc is significantly disturbed under Fe deprived conditions, while binding of Grx4-GFP and Fep1-Myc is constitutive (Fig. 1F). These results suggest that the interaction of Grx4 with Php4, but not with Fep1, is partially disturbed upon Fe starvation.

### The glutaredoxin Grx4 is a Fe-S cluster-containing protein

**Fig 2:**
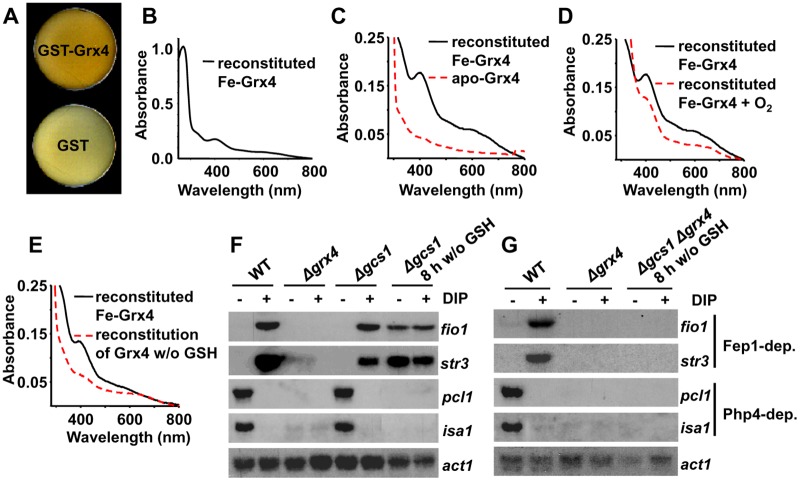
The glutaredoxin Grx4 is a Fe-S cluster-containing protein. (A) Cells pellets of *E*. *coli* over-expressing GST-HA-Grx4 are brown. Cells transformed with plasmids pGEX-2T-TEV (GST) or p400 (pGEX-2T-TEV-GST-*HA-grx4*; GST-Grx4) were grown into LB, and protein expression was induced by IPTG. Cells were pelleted on Elisa plates and photographed. (B and C) Reconstitution of the Fe-S cluster of Grx4. UV-visible absorption spectra of reconstituted Fe-HA-Grx4 (Fe-Grx4). The red dashed line indicates the spectrum of the apo-protein, obtained in the absence of added Fe. (D) The reconstituted Fe-S cluster of Grx4 is sensitive to oxygen. UV-visible absorption spectra of reconstituted Fe-HA-Grx4 (Fe-Grx4) protein before (solid line) and after (red dashed line) 15 minutes of oxygen exposure. (E) GSH is required for reconstitution of the Grx4 Fe-S cluster. UV-visible absorption spectra of Grx4 reconstitution reactions performed in the presence (solid line) or absence (red dashed line) of GSH. (F) Cell lacking Gcs1, auxotrophic for GSH, display constitutive activation of the Fe starvation response after 8 h of GSH withdrawal. Strains 972 (WT), NG81 (*Δgrx4*) and NG77 (*Δgcs1*) were grown in YE media, and shifted to MM without GSH, when DIP was added. When indicated for strain NG77, growth proceeded for 8 h prior to DIP addition. Total RNA was analyzed as described in Fig. 1E. (G) Same as in F, with strains 972 (WT), NG81 (*Δgrx4*) and JE7 (*Δgcs1 Δgrx4*).

Mammalian Grx2, a nuclear-mitochondrial glutaredoxin, was the first thioredoxin fold-containing protein reported to have a Fe-S cluster [19]. More recently, the redundant monothiol Grx3/4 glutaredoxins of *S*. *cerevisiae* were also reported to be Fe-S cluster-containing proteins, and to use two glutathione (GSH) moieties to hold the cluster [10,12,13]. We over-expressed a TEV-cleavable GSH-S-transferase (GST)-TEV-HA-Grx4 fusion protein in *Escherichia coli*, and noticed than the cell pellets (Fig. 2A) and the early supernatants had brownish color when compared with bacteria over-expressing GST alone. The color disappeared during protein purification. By comparing tagged and un-tagged proteins, we verified that the HA tag did not affect the properties of recombinant Grx4. We hypothesized that Grx4 can assemble an oxygen-sensitive Fe-S cluster, and attempted to reconstitute it under anaerobic conditions. We incubated recombinant TEV-cleaved apo-HA-Grx4 with Fe, inorganic sulfide and GSH in the presence of the *E*. *coli* Fe-S cluster catalyzer IscS [20]. As observed by UV-visible spectroscopy, two shoulders in the 390–650 nm regions could be detected after Fe-S cluster reconstitution, with an apparent extinction coefficient at 398 nm of 3.2 mM^-1^ cm^-1^ (Fig. 2B,C); the samples lost, although not completely, these visible spectra peaks after oxygen exposure (Fig. 2D) and became colorless. GSH was required for Fe-S cluster reconstitution (Fig. 2E), which suggests that the tripeptide coordinates the Fe-S cluster, as previously shown for other monothiol glutaredoxins.

To corroborate the relevance of GSH in cluster formation and in the role of Grx4 in Fe sensing, we analyzed the transcriptome of strain *Δgcs1* in response to Fe deprivation. Cells lacking *gcs1*, coding for glutamate-Cys ligase [21], are able to grow in GSH-containing rich media, but cultures halt their growth few hours after cells are shifted to minimal media, as expected. Under these circumstances, the transcriptome of *Δgcs1* cells displays constitutive repression of Php4-genes and constitutive de-repression of Fep1-dependent genes (Fig. 2F); this last feature is completely dependent on the presence of Grx4 (Fig. 2G). These results suggests that GSH is required to allow the assembly of an Fe-S cluster in Grx4, and that this cluster is essential for the function of Grx4 as a Fe deprivation sensor and signal transducer.

### The Fe-S cluster of Grx4 is essential for both Fe delivery and Fe signaling

**Fig 3:**
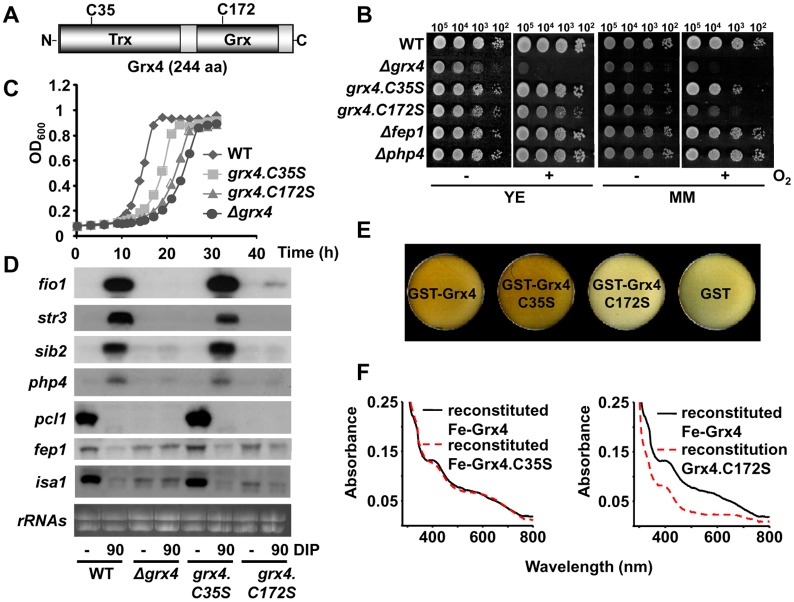
The Fe-S cluster of Grx4 is essential for both Fe delivery and Fe sensing. (A) Scheme of the 244 aa-long Grx4 protein, showing the conserved Cys-containing thioredoxin (Trx) and glutaredoxin (Grx) domains. (B and C) Cells expressing Grx4.C172S display growth defects under aerobic conditions. (B) Survival spots of cultures from strains 972 (WT), NG81 (*Δgrx4*), NG86.C35S (*grx4*.*C35S*), NG86.C172S (*grx4*.*C172S*), NG2 (*Δfep1*) and NG40 (*Δphp4*), as described in Fig. 1B. (C) Growth curves of 972 (WT), NG81 (*Δgrx4*), NG86.C35S (*grx4*.*C35S*) and NG86.C172S (*grx4*.*C172S*) strains were recorded as described in Fig. 1C. (D) Total RNA from strains 972 (WT), NG81 (*Δgrx4*), NG86.C35S (*grx4*.*C35S*) and NG86.C172S (*grx4*.*C172S*) was processed as described in Fig. 1E. (E) Cells pellets of *E*. *coli* over-expressing GST-Grx4.C172S are not brown. Cells transformed with plasmids pGEX-2T-TEV (GST), p400 (pGEX-2T-TEV-*HA-grx4*; GST-Grx4), p400.C35S (pGEX-2T-TEV-*HA-grx4*.*C35S*; GST-Grx4.C35S) or p400.C172S (pGEX-2T-TEV-*HA-grx4*.*C172S*; GST-Grx4.C172S) were grown and cell color analyzed as described in Fig. 2A. (F) UV-visible absorption spectra of wild-type and mutant Grx4 after Fe-S cluster reconstitution.

Grx4 has one Cys residue in the thioredoxin domain and another one in the glutaredoxin domain (Fig. 3A). We substituted the endogenous *grx4* locus with mutant versions with a Cys-to-Ser codon substitution in either the thioredoxin or the glutaredoxin domains. Fission yeast cells expressing Grx4.C35S behaved very similar to wild-type cells regarding both aerobic growth (Fig. 3B,C) and activation of the transcriptional Fe starvation response (Fig. 3D). On the contrary, Grx4.C172S was unable to fulfill any function of Grx4, since cells expressing this mutant protein display phenotypes very similar to *Δgrx4* cells (Fig. 3B,C,D). It is important to point out that the *grx4*.*C172S* strain was isolated under semi-anaerobic conditions.

We next over-expressed TEV-cleavable GST-(TEV)-HA-Grx4.C35S and C172S fusion proteins in *E*. *coli*, and noticed again than the cell pellets for the wild-type and Grx4.C35S tagged proteins were clearly brownish, while those of cells over-expressing the Grx4.C172S fusion protein were colorless and similar to those of bacteria over-expressing GST alone (Fig. 3E). Again, we attempted to reconstitute the oxygen-sensitive metallocenters under anaerobic conditions. As shown in Fig. 3F (left panel), reconstitution of recombinant Grx4.C35S yielded a protein with similar visible spectrum to that of wild-type Fe-Grx4. However, the presence of Cys172 was required for cluster assembly *in vitro*, since reconstitution could not be observed for mutant Grx4.C172S (Fig. 3F, right panel). These results suggest that the Fe-S cluster of Grx4 is essential for both functions: aerobic growth and Fe signaling via Php4 and Fep1.

### The BolA-like protein Fra2 is required for Grx4- and Fep1-dependent de-repression of transcription

**Fig 4:**
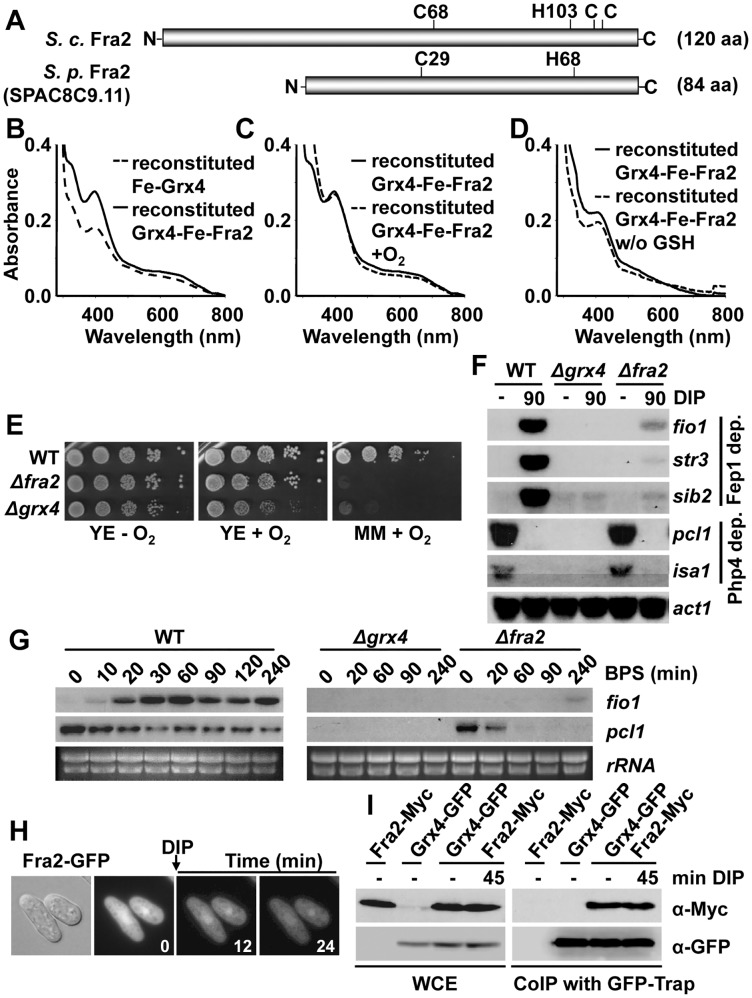
The BolA protein Fra2 participates in the Grx4-dependent signaling cascade. (A) Scheme of the 84 aa-long Fra2 protein, showing the conserved Cys and histidine residues important in the *S*. *cerevisiae* homolog. (B to D) Reconstitution of a Fe-S cluster bridging Grx4 and Fra2. (B) UV-visible absorption spectra of reconstituted Fe-Grx4 (dashed line) or Grx4-Fe-Fra2 (solid line) proteins. (C) UV-visible absorption spectra of reconstituted Grx4-Fe-Fra2 protein before (solid line) and after (dashed line) 15 minutes of oxygen exposure. (D) UV-visible absorption spectra reconstitution of Grx4-Fe-Fra2 in presence (solid line) or absence (dashed line) of GSH. (E) Cells lacking Fra2 display growth defects under aerobic conditions. Strains 972 (WT), NG101 (*Δfra2*) and NG81 (*Δgrx4*) were spotted and grown on YE and MM plates under aerobic or anaerobic conditions. (F) The Fep1-dependent gene expression program is compromised in cells lacking Fra2 in response to DIP. Total RNA from strains 972 (WT), NG101 (*Δfra2*) and NG81 (*Δgrx4*) was processed as described in Fig. 1E. (G) Fra2 is not fully dispensable in the Php4-dependent Fe starvation response in response to BPS. Total RNA was obtained from YE cultures of strains 972 (WT), NG101 (*Δfra2*) and NG81 (*Δgrx4*), treated or not with 25 μM BPS for the indicated times in minutes, and analyzed by Northern blot with the probes indicated. *rRNA* was used as a loading control. (H) Fra2 displays both cytosolic and nuclear localization. Strain JE3 (*fra2-GFP*) was analyzed by fluorescence microscopy before and after DIP treatment. (I) Fra2 interacts with Grx4 *in vivo*. Strains JE5 (*fra2-myc*), NG115 (*grx4-GFP*) and JE17 (*grx4-GFP fra2-myc*) were analyzed as described in Fig. 1F.

The Fe-S cluster of the redundant Grx3/4 proteins in *S*. *cerevisiae* was also reported to be oxygen-sensitive [14]. The cluster was more stable if the protein was co-expressed in bacteria with Fra2, a protein originally shown at the genetic level to be required to transduce an Fe starvation signal to the yeast transcriptional activator Aft1 [14,15,22]. The *S*. *pombe* genome has an *S*. *cerevisiae’s fra2* homolog, *SPAC8C9*.*11* (Fig. 4A). We performed *in vitro* reconstitution of the Grx4 Fe-S cluster in the presence of equimolar recombinant Fra2, yielding a coloured sample with different UV-visible spectrum (apparent ε_398_: 5.0 mM^-1^ cm^-1^) to that formed within a Grx4 homodimer, suggesting the formation of an Fe-S cluster bridging Grx4 and Fra2 (Fig. 4B, solid line). This cluster was not sensitive to the presence of oxygen (Fig. 4C). The presence or absence of GSH in the Grx4-Fe-Fra2 reconstitution process only moderately affected the resulting visible spectra (Fig. 4D). Indeed, *in vitro* studies with the *S*. *cerevisiae* GRX4-FRA2 heterodimer suggest that only one GSH molecule, and not two, is present in the holo-heterodimer [14]. The Cys172 in the glutaredoxin domain, but not Cys35, of Grx4 is important for the assembly of the Fe-S cluster between Grx4 and Fra2 (S3A Fig).

We constructed a *Δfra2* strain, and observed that it also displays aerobic growth defects, although of less severity that cells lacking Grx4: in the absence of Fra2, cells grow as wild-type on YE plates, but the growth is strongly impaired on respiratory-prone MM plates, where many Fe-containing proteins mediate redox reactions (Fig. 4E). With regards to Fe deprivation, *Δfra2* cells are only mildly sensitive to iron chelators such as DIP or BPS on solid plates (S3B Fig), and the growth in liquid cultures is only moderately affected by these chelators, when compared to cells lacking Grx4 (S3C Fig).

Regarding the transcriptional response to Fe deprivation, cells lacking Fra2 are able to repress Php4-dependent genes upon DIP treatment as wild-type cells, but cannot efficiently induce the Fep1-dependent Fe uptake genes (Fig. 4F). Therefore, Php4 can be retained in the cytosol by Grx4 in the absence of Fra2 under Fe repleted conditions, but inactivation of Fep1 upon addition of DIP requires both Grx4 and Fra2. Interestingly, the use of BPS as a chelator revealed a marginal role of Fra2 not only in Fep1 regulation but also in Php4 cytoplasmic retention during normal growth conditions: in cells lacking Fra2, activation of Php4 as determined by repression of *pcl1* is much faster and dramatic that in wild type cells, which suggest that Grx4-Fe-Fra2 is able to retain Php4 in the cytosol with more efficiency than Fe-Grx4 alone (Fig. 4G).

The role of Fra2 on Fep1 function is not to promote the association between Grx4 and Fep1, since this interaction is maintained in cells lacking Fra2 (S3D Fig). Similar to Grx4, Fra2 displays dual cytoplasmic/nuclear localization that is not affected by treatment with DIP, according to fluorescence microscopy (Fig. 4H). Indeed, both proteins constitutively interact as shown by co-immuno-precipitation (Fig. 4I). It is worth mentioning that during the course of this study, the group of Labbé proposed that *S*. *pombe* Fra2 participates with Grx4 in the inactivation of the Fep1 repressor [23].

### Fep1 is also a Fe-containing protein

Activation of Php4 upon Fe starvation seems to be straightforward: addition of chelators probably promotes Fe-S cluster disassembly from Grx4-Fra2, and the apo-protein may lose affinity for Php4, which is then accumulated at the nucleus. Grx4 is also important for Fep1 function, but contrary to what happens for Php4-dependent genes, the levels of Fep1-dependent ones in *Δgrx4* cells do not mimic a Fe-starvation situation; instead, they are constitutively repressed (Fig. 1E). Similarly, Grx4.C172S should mimic a Fe starvation situation, but the transcriptome of cells expressing this mutant protein reveals that only Php4 genes are fully repressed, and on the contrary Fep1 genes cannot be activated (Fig. 3D). How is then the Fe starvation signal transferred to Fep1?

**Fig 5:**
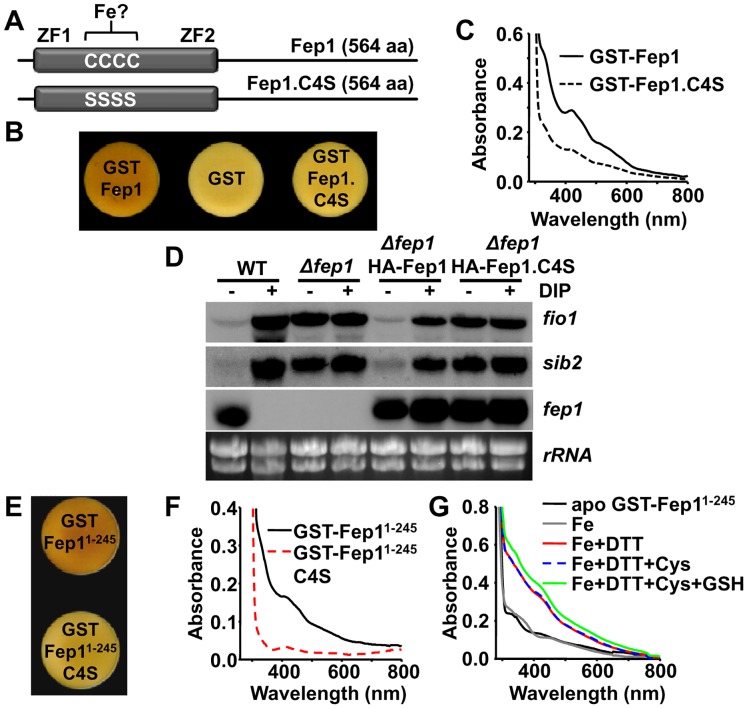
The Fep1 repressor is also a Fe-containing protein. (A) Scheme of the 564 aa-long Fep1 protein, showing the four Cys residues between the zinc fingers (ZF1 and ZF2) which were mutated to serine residues (Fep1.C4S). (B) Cells pellets of *E*. *coli* over-expressing GST-Fep1, but not GST-Fep1.C4S, are brown. Cells transformed with plasmids pGEX-2T-TEV (GST), p514 (pGEX-2T-TEV-*fep1;* GST-Fep1) or p514.C4S (pGEX2T-TEV-*fep1*.*C4S;* GST-Fep1.C4S) were grown and cell color analyzed as described in Fig. 2A. (C) UV-Visible spectra of GST-Fep1 (solid line) or GST-Fep1.C4S (dashed line). (D) The Fep1-dependent gene expression program is compromised in cells expressing HA-Fep1.C4S. Total RNA from strains 972 (WT), JE16 (*Δfep1*) alone or transformed with episomal plasmids p516.81x or p516.81x.C4S (allowing the expression of HA-Fep1 or HA-Fep1.C4S under the control of the weak *nmt* promoter) was obtained from MM cultures after 18 h thiamine withdrawal, and analyzed as described in Fig. 1E. (E) Cells pellets of *E*. *coli* over-expressing GST-Fep1^1–245^, but not GST-Fep1^1–245^.C4S, are brown. Cells containing p514.NTD (pGEX-2T-TEV-*fep1* ^*1–245*^ *;* GST-Fep1^1–245^) or p514.NTD.C4S (pGEX-2T-TEV-*fep1* ^*1–245*^.*C4S;* GST-Fep1^1–245^.C4S) were grown and cell color analyzed as described in Fig. 2A. (F) UV-Visible spectra of GST-Fep1^1–245^ (solid line) or GST-Fep1^1–245^.C4S (red dashed line) proteins. (G) A sulfur donor (L-Cys) is not required for reconstitution of the GST-Fep1^1–245^ cluster. UV-visible absorption spectra after reconstitution reactions of apo GST-Fep1^1–245^ (solid black line) in the presence of Fe (Fe; solid grey line), Fe and DTT (solid red line), Fe, DTT and L-Cys (dashed blue line) or the standard reconstitution reaction with Fe, DTT, L-Cys and GSH (solid green line).

Fep1 belongs to the GATA family of transcriptional repressors (for a review, see [24]). It contains two zinc finger motifs for DNA binding at the N-terminal domain, flanking a Cys-rich region with four important Cys residues, which when mutated completely disturbed *in vitro* and *in vivo* DNA binding and gene repression, respectively [6] (Fig. 5A). Furthermore, the ability of recombinant Fep1 to bind to DNA *in vitro* was greatly diminished when the protein was purified from Fe-starved cultures [2]. We over-expressed a TEV-cleavable GST-Fep1 fusion protein in *E*. *coli*, and again the cell pellets had brownish color when compared with bacteria over-expressing GST alone (Fig. 5B). The GST-Fep1 protein, before (Fig. 5C, solid line) or after TEV cleavage, retained the brown color during purification, and displayed a characteristic visible spectrum. Multiple Cys-to-Ser substitutions at the Cys-rich domain fully abrogated the brownish color of cells over-expressing the transcription factor (Fig. 5B), and flattened the visible spectrum of the purified protein (Fig. 5C). When HA-tagged Fep1 was expressed in *S*. *pombe Δfep1* cells from an episomal plasmid, the tagged protein was able to repress Fe import genes under basal conditions, whereas mutated HA-Fep1.C4S could not (Fig. 5D). Therefore, binding of Fe by Fep1 seems to be required for its role as a transcriptional repressor.

The GST-tagged full length Fep1 protein was however very susceptible to degradation, and we therefore purified a more stable, shorter GST fusion protein containing only the first 245 N-terminal amino acids (S4A Fig): this truncated protein still retained Fe, as determined both *in vivo* (Fig. 5E) and as a purified protein (Fig. 5F). This N-terminal Fep1^1–245^ protein still relies on the four Cys residues for metal binding (Fig. 5E,F), and is more stable than the full length protein.

In order to determine whether Fep1 is an Fe-S protein or it just directly coordinates Fe, we determined the stoichiometry of Fe-to-protein and inorganic sulfide-to-protein, using a *bona fide* Fe-S cluster containing protein, the *E*. *coli* SoxR transcription factor, as a control [25]. Thus, we purified SoxR (S4A Fig), which displayed the characteristic UV-visible spectrum of its [2Fe-2S] cluster (S4B Fig) [25], and determined a 0.9:1 ratio for both Fe and acid labile sulfide (Table 1) to SoxR monomer. On the contrary, we measured a 1:1 Fe-to-protein ratio in purified GST-Fep1^1–245^, which was dependent on the presence of the four Cys residues at the N-terminal domain, but could not detect inorganic sulfide (Table 1). This suggests that the Fe is not bound to Fep1 in the form of a Fe-S cluster.

**Table 1:** Fe and acid labile sulphide content of partially purified recombinant proteins (all concentrations in μM).

**Fe content**				
**Preparation**	**1**	**2**	**3**	**Fe to protein**
**SoxR**	35	41	37	0.93 ± 0.07
**Fe**	30.4	41.3	34.6	
**GST-Fep1** ^1–245^	28	19	24	1.04 ± 0.19
**Fe**	22.6	22.6	23.1	
**GST-Fep1** ^1–245^ **.4S**	34	13.5	17	0.14 ± 0.09
**Fe**	3.2	3.1	1.1	
**Labile sulphide content**				
**Preparation**	**1**	**2**	**3**	**Sulfide to protein**
**SoxR**	35	38	32	0.93 ± 0.04
**Sulfide**	34	35	28.7	
**GST-Fep1** ^1–245^	39	45	55	0.09 ± 0.01
**Sulfide**	3	4.8	5.1	
**GST-Fep1** ^1–245^ **.4S**	41	52	83	0.03 ± 0.02
**Sulfide**	0.45	2.9	3.2	

To confirm this result, we obtained an apo form of Fep1 upon purification of GST-Fep1^1–245^ in thiol-containing buffers (1 mM β-mercaptoethanol), and managed to recover *in vitro* the peaks of absorption of the Fe-containing protein upon anaerobic incubation with Fe and reducing agents, in the absence of a sulfide donor (Fig. 5G, red solid line). This result suggests that Fe is bound to Fep1 directly to the Cys residues located between the zinc fingers, and not as a Fe-S cluster.

**Fig 6:**
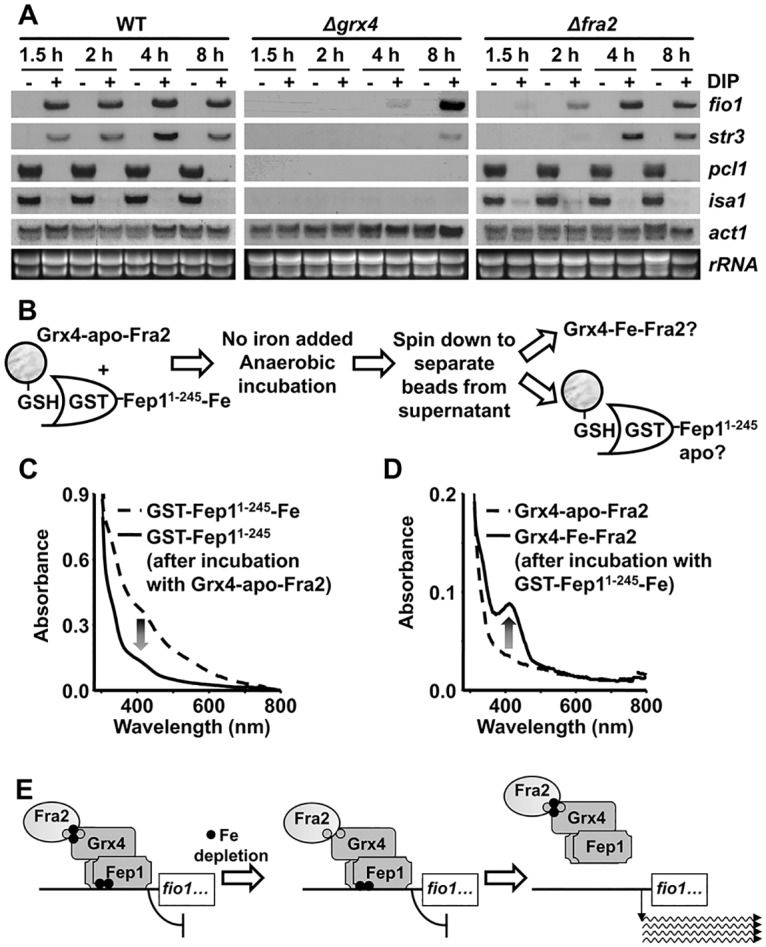
Reverse Fe transfer between Grx4-apo-Fra2 and Fe-containing Fep1 followed by UV/visible spectroscopy. (A) Upon long DIP treatments, Grx4 and Fra2 are dispensable for activation of Fep1-dependent genes. Total RNA from YE cultures of strains 972 (WT), NG81 (*Δgrx4*) and NG101 (*Δfra2*), before and after the indicated time in hours with DIP, was processed as described in Fig. 1E. (B) Scheme of the metal transfer reaction between GST beads-bound GST-Fep1^1–245^ and Grx4-apo-Fra2. (C and D) *In vitro* Fe transfer from Fe-Fep1 to apo-Grx4-Fra2. UV/visible spectra of GST-tagged Fep1 (C) and Grx4-Fra2 (D) were recorded before (dashed line) and after (solid line) incubation in a 1:1 protein ratio and protein separation through GSH-affinity chromatography. (E) Model proposed for the reverse metal transfer reaction between Fe (solid circles)-containing Fep1 and Fe-depleted Grx4-Fra2. See text for details.

Our results indicate that Fep1 is a Fe-containing protein. Since the Fep1.C4S mutant, unable to bind Fe *in vitro*, displays de-repressed gene transcription (Fig. 5D), a simple hypothesis is that intracellular Fe depletion could then render apo-Fep1 and activation of Fe import genes. Why are then Grx4 and Fra2 required for this de-repression? We explored the possibility that these proteins are just facilitators of the loss of Fe from Fe-Fep1, where the metal should be tightly bound to the protein backbone. Indeed, under longer DIP chelator treatments we observed de-repression of Fep1-dependent genes even in the absence of Grx4 (8 hours; Fig. 6A) or Fra2 (2–4 hours; Fig. 6A). In *S*. *cerevisiae*, Fe-GRX3/GRX4 participates in the Fe-S cluster assembly pathway, where it probably transfers its iron-sulfur cluster towards the protein NAR1, a protein involved in the assembly of cytosolic and nuclear iron-sulfur proteins (for a review, see [26]. Our *in vivo* data suggest that Grx4-Fra2 may be ‘facilitators’ in the loss of Fe by Fep1, by promoting a ‘reverse’ Fe transfer reaction, as opposed to the proposed role of this complex in iron-sulfur cluster assembly [26].

We then design an *in vitro* approach to investigate reverse Fe transfer from Fep1 to Grx4-Fra2. We performed reconstitution of the Fe-S cluster of Grx4-apo-Fra2 under anaerobic conditions in the absence of inorganic Fe and with the only metal supply of Fe-containing GST-Fep1^1–245^ (Fig. 6B). After 2 hours of incubation the UV-visible spectrum shoulders of Fe-containing Fep1 were lost (Fig. 6C), concomitant to the assembly of the Fe-S cluster to yield Grx4-Fe-Fra2 (Fig. 6D). Importantly, this reverse metal transfer process is abolished if apo-GST-Fep1.C4S (S5A Fig) or Grx4.C172S (S5B Fig) are components of the reaction. Furthermore, we could not detect Fe transfer from Grx4-Fe-Fra2 to apo-GST-Fep1^1–245^ using the same experimental conditions (S5C Fig). We propose that upon Fe loss from the Fe-S cluster bridging Grx4 and Fra2, the heterodimer can then sequester in a reverse transfer process the Fe from Fep1, which would then become inactive as a transcriptional repressor (Fig. 6E).

## Discussion

Regulation of the intracellular Fe pools largely depends on the activation of Fe uptake and inhibition of Fe storage and consumption during Fe starvation. In *S*. *pombe*, this process fully depends on Grx4, a monothiol glutaredoxin reported in other eukaryotic organisms to regulate both Fe traffic and Fe signaling events. We have generated *grx4* and *fra2* knock out strains, and mutants integrated at the chromosomal *grx4* locus, to unambiguously determine their participation in both processes. We demonstrate that Grx4-Fra2 is a Fe-S cluster-containing heterodimer, which is essential for both Fe delivery and Fe sensing/signaling. Grx4-Fra2 is localized at the cytosol and the nucleus before and after Fe starvation. Addition of chelators to the cell cultures triggers a Fe starvation response, probably by eliminating the Fe-S clusters in the Grx4-Fra2 dimers. However, the molecular events regulating Php4 and Fep1 are quite distinct.

Php4 is the only component of this complex Fe signaling cascade that is regulated at the level of subcellular localization: it is retained at the cytosol under rich Fe conditions in a Grx4-dependent manner. Under Fe deprivation, the association of Grx4-Fra2 to Php4 is significantly disturbed, as demonstrated here by co-immuno-precipitation analysis (Fig. 1G), and Php4 then accumulates at the nucleus, where it represses Fe usage genes. Fra2 was recently described to be fully dispensable for the Php4-dependent regulation of transcription in response to the permeable Fe chelator DIP [23], but we show here that in *Δfra2* cells the kinetics of Php4 activation as a repressor in response to the mild chelator BPS are much faster than in wild-type cells (Fig. 4G). We propose that Fe-containing Grx4 homodimers can retain Php4 in the cytosol but with less efficiency than Grx4-Fe-Fra2, and therefore BPS can more easily achieve Php4 activation in a *Δfra2* background than in wild-type cells. The loss of Fe from the Grx4-Fra2 dimers seems to be the triggering event: our *in vitro* reconstitution experiments demonstrate that Cys172 in Grx4 is required for cluster assembly in the homo (Fig. 3F) or heterodimer (S3A Fig), and cells expressing only Grx4.C172S display a transcriptional program to Fe deprivation identical to that of cells lacking Grx4, with constitutive repression of Php4-dependent genes (Fig. 3D). We propose that Fe-containing Grx4-Grx4 or, to a better extent, Grx4-Fra2 complexes are capable of binding to and sequestering Php4 in the cytosol, and that Fe-S cluster loss either by Fe starvation of by expression of the constitutive apo-form Grx4.C172S render cells with constitutively nuclear Php4.

The molecular events ruling the Grx4-dependent Fep1 activity are not so straightforward. Instead of displaying constitutive transcription of Fe import genes, cells expressing Grx4.C172S cannot activate them in response to Fe deprivation (Fig. 3D). Thus, loss of metal binding in Grx4 upon Fe starvation cannot be sufficient for Fep1 inactivation as a repressor. We show here that Fep1 is also a Fe-binding protein, as demonstrated by characterization of recombinant GST-Fep1 (Fig. 5C and Table 1). The presence of Fe in other recombinant GATA repressors, such as SRE of *Neurospora crassa* [27] or Sre1 of the pathogenic fungus *Histoplasma capsulatum* [28], has already been described. Fep1 has some Cys residues of the N-terminal domain essential for metal binding (Fig. 5B,C and Table 1) and transcriptional repression activity (Fig. 5D). This Fe is probably tightly bound to the protein: while moderate Fe decreases (i.e. 1.5 h long DIP treatments) are not sufficient to withdraw the metal from Fep1 in the absence of Grx4 or Fra2 and de-repress transcription, longer chelator exposures (2 or 8 h) are able to accomplish it in *Δfra2* or *Δgrx4* strains, respectively (Fig. 6A).

Which is then the role of the Grx4-Fra2 heterodimer in Fep1 inactivation as a repressor? We propose that the Fe-S cluster in the Grx4-Fra2 complex may be partially or totally dismantled upon metal starvation, and the Fe-lacking Grx4-Fra2 can then induce reverse metal transfer from Fep1 (Fig. 6C,D), as recently proposed *in vitro* for the monothiol glutaredoxin Grx3-Fra2 heterodimer and the downstream Fe-S cluster carrier proteins Isc1 [29]. We also suggest that the Fe-S cluster between Grx4 and Fra2 is specifically important to bridge these two proteins, but not for binding to and inactivating Fep1. This is supported by the following facts: (i) the cluster between Grx4.C172S and Fra2 cannot be reconstituted *in vitro* (S3A Fig); (ii) the *in vivo* binding between Grx4 and Fep1 is only mildly affected in cells expressing Grx4.C172S-GFP (S6A Fig); and (iii) the kinetics of activation of Fe import genes upon DIP in cells lacking Fra2 are almost identical to those of cells expressing the Grx4.C172S mutant (compare Fig. 6A with S6B).

The fact that Grx4 regulates by different mechanisms two transcriptional repressors is intriguing. With the use of the chelator BPS, which naturally depletes extracellular Fe and which therefore acts indirectly on the intracellular Fe levels, it has been surprising to detect important differences in the kinetics of regulation of Fep1- and Php4-dependent genes. Thus, activation of genes coding for Fe importers occurs immediately, whereas repression of Fe usage genes is not as fast and it is less pronounced as in response to DIP (Fig. 1D). Therefore, and at least after BPS exposure which seems to induce a more physiological Fe starvation than DIP, fission yeast attempts to induce Fe import as a first wave of action, with only a mild down-regulation of Fe storage and consumption. This hierarchical activation of Fe import prior to repression of Fe usage may be a rather general strategy of cells upon Fe starvation: at least in *S*. *cerevisiae*, the expression of Cth2, a protein which promotes degradation of mRNAs encoding Fe-containing proteins, depends at the level of transcription on Aft1, a transcription factor which responds to Fe deprivation and triggers transcription of Fe import genes, and therefore occurs later on time than the activation of Fe import [30].

Cells lacking Grx4 or, to a lesser extent, Fra2 display severe growth defects in the presence of oxygen that are not shared by cells lacking Php4 or Fep1 (Fig. 1B and4E). This fact prompts us to speculate that *S*. *pombe* Grx4 has an essential role in Fe delivery towards Fe-containing proteins, as it has been proposed for the *S*. *cerevisiae* homolog GRX4 [10]. Some of the aerobic phenotypes of cells lacking Grx4 could arise from the constitutive repression of Php4-dependent genes (Fig. 1E, right panel), most of which are essential for respiratory growth [8]. We have, however, dismissed this possibility with the characterization of cells lacking both Grx4 and Php4: the expression of respiratory-related genes is wild-type in this cell background under normal Fe conditions, and nevertheless cells are still defective to grow under aerobic conditions (S7A–S7B Fig). Another fact that supports the idea of Grx4 and Fra2 participating in a process other than sensing Fe depletion to activate signaling cascades is the intracellular concentrations of these components. Thus, while Php4 and Fep1 are present in 1–3 thousand copies per cell, the concentration of Grx4 and Fra2 has been described to be in the order of 19 and 16 thousand copies per cell, respectively, according to a recent proteomic report [31]. We have confirmed these 5–15-fold higher concentrations of Grx4 and Fra2 with respect to the transcriptional repressors Fep1 and Php4 with C-terminal *myc* tagging of the four gene loci and immuno-blotting (S7C Fig). Contrary to what has been proposed for *S*. *cerevisiae*, not only Grx4 but also Fra2 may contribute to Fe delivery to downstream effectors in the Fe-S cluster assembly pathway in *S*. *pombe*. Whether general Fe-containing proteins are defective in Fe content and activity in *Δgrx4 Δphp4* cells, where the concentrations of Fe-containing proteins are as in wild-type cells, will have to be analyzed.

## Materials and Methods

### Strains, plasmids and growth conditions

Origins and genotypes of strains used in this study are outlined in S1 Table. Details on their construction and growth conditions, as well as on plasmids construction, are provided in S1 Text.

### Solid sensitivity assay and growth curves

For survival on solid plates, *S*. *pombe* strains were grown in YE, diluted and spotted in YE or MM medium agar plates as described previously [32]. Growth curves were also measured as previously described [32]. Details are provided in S1 Text.

### RNA analysis

Total RNA from exponentially growing *S*. *pombe* cells in YE, with or without treatment with the Fe chelators DIP (0.1 or 0.25 mM) or BPS (25 μM), was extracted, processed and analyzed as previously reported [33].

### Immuno-fluorescence assay

Cells were fixed with formaldehyde, treated with zymolyase in the presence of sorbitol, and resulting spheroplasts were incubated with polyclonal anti-Grx4, or monoclonal anti-HA (12CA5) antibodies. After incubation with corresponding secondary antibodies, cells were analyzed directly by fluorescence microscopy as described previously [34]. Details are provided in S1 Text.

### Fluorescence microscopy

Fluorescence microscopy and image capture was performed as previously described [34].

### Co-immunoprecipitation analysis

Analysis was performed as previously described [35].

### Bacteria growth conditions, analysis of the color of cell pellets, and purification of recombinant proteins

Bacteria strain FB810 [36] transformed with the pGEX-2T-TEV derivatives were grown at 18°C for efficient protein expression as described in S1 Text. Analysis of the color of cell pellets, purification of GST-tagged proteins with glutathione-sepharose beads and cleavage with Tev protease is also described in S1 Text, as well as purification of the untagged control bacterial protein SoxR.

### Fe-S cluster reconstitution assay and Fe transfer reaction assay

Cluster reassembly and Fe transfer reactions were performed under anaerobic conditions in a Forma Anaerobic System (Thermo Electron Corporation). Details on the iron reconstitution reactions of recombinant wild-type or mutant Grx4 (50–60 μM) with or without equimolar amounts of Fra2, or GST-Fep1^1–245^, are detailed in S1 Text, as well as the Fe transfer reactions between Fe-GST-Fep1^1–245^ and apo-Grx4-Fra2.

### Colorimetric assays for Fe and acid labile sulfide quantification

Fe [37] and acid labile sulfide [38] quantification was performed as previously described with the modifications indicated in S1 Text.

### Preparation of S. pombe trichloroacetic acid (TCA) extracts and Western blot to measure protein concentration

TCA extracts and Western blot was performed as previously described [39].

## Supporting Information

S1 Fig Effect of different Fe chelators in the growth of fission yeast. (A) Growth curves of wild-type strain in the presence or absence of the indicated concentrations of chelators. Log-phase culture (OD_600_: 0.1) of the wild-type strain 972 was treated or not with the indicated concentrations of DIP, Dx or BPS, and grown into microculture wells. Growth was monitored by measuring OD_600_ every 10 min at 30° for 48 h. (B) Cells lacking Php4 or Grx4 display growth defects in the presence of different Fe chelators. Strains 972 (WT), NG2 (*Δfep1*), NG40 (*Δphp4*) and NG81 (*Δgrx4*) were spotted and grown under anaerobic conditions on plates containing the indicated concentrations of Dx, DIP or BPS.
(TIF)

S2 Fig Cellular localization of Grx4, Fep1 and Php4. (A and B) Only Php4, but not Grx4 or Fep1, changes its sub-cellular localization upon Fe starvation. (A) Cellular localization of untagged Grx4, Php4-HA and Fep1-HA, before or after 90 min treatment with DIP, was determined by immuno-fluorescence microscopy from strains 972 (WT), NG64 (*fep1-HA*) and NG123 (*php4-HA*). (B) Cellular localization of GFP-tagged Grx4, Php4 and Fep1 was determined by fluorescence microscopy from strains NG115 (*grx4-GFP*), NG105 (*fep1-GFP*) and NG70 (*php4-GFP*), before and after treatment with DIP for the times indicated.
(TIF)

S3 Fig Role of Fra2 in the Grx4-dependent Fe starvation response. (A) Reconstitution of the Fe-S cluster of Grx4-Fra2. Electrophoretic analysis of recombinant proteins obtained in *E*. *coli*, BSA (0.5 and 1 μg) was used as a loading control. UV-visible absorption spectra of reconstituted Grx4-Fe-Fra2 (solid line), reconstituted Grx4.C35S-Fe-Fra2 (red dashed line) or reconstitution reaction with Grx4.C172S-Fra2 (black dashed line). (B) Cells lacking Fra2 display minor defects in the presence of Fe chelators such as Dx, DIP or BPS. Strains 972 (WT), NG101 (*Δfra2*) and NG81 (*Δgrx4*) were spotted and grown under aerobic conditions on YE plates containing or not the indicated concentrations of chelators. (C) Growth curves of wild-type, *Δgrx4* and *Δfra2* strains in the presence or absence of chelators. Growth of strains 972 (WT), NG101 (*Δfra2*) and NG81 (*Δgrx4*) was monitored as indicated in S1A Fig. (D) The *in vivo* interaction between Grx4 and Fep1 is not disturbed in cells lacking Fra2. Co-immunoprecipitation assays in extracts from strains NG115 (WT *grx4-GFP*), NG108 (WT *fep1-myc*), NG109 (WT *grx4-GFP fep1-myc*), JE6 (*Δfra2 grx4-GFP*), JE8 (*Δfra2 fep1-myc*), and JE4 (*Δfra2 grx4-GFP fep1-myc*) was performed as described in Fig. 1F.
(TIF)

S4 Fig Purification of recombinant SoxR and GST-Fep1. (A) Electrophoretic analysis of recombinant proteins obtained in *E*. *coli*. BSA was used as a loading control. (B) UV-Visible spectrum of purified bacterial SoxR protein.
(TIF)

S5 Fig Reverse Fe transfer is impaired in the mutants Grx4.C172S and GST-Fep1^1–245^.C4S. (A) UV-visible spectra of GST-tagged Fep1^1–245^.C4S and Grx4-Fra2 were recorded before (dashed line) and after (solid line) incubation in a 1:1 protein ratio and protein separation through GSH-affinity chromatography. (B) UV-visible spectra of GST-tagged Fep1^1–245^ and Grx4.C172S-Fra2 were recorded before (dashed line) and after (solid line) incubation in a 1:1 protein ratio and protein separation through GSH-affinity chromatography. (C) GST-Grx4-Fe-Fra2 cannot transfer Fe to apo-Fep1^1–245^ in our *in vitro* system. UV/visible spectra of GST-tagged apo-Fep1^1–245^ (left) or GST-Grx4-Fe-Fra2 (right) were recorded before (dashed line) and after (solid line) incubation in a 1:1 protein ratio and protein separation through GSH-affinity chromatography.
(TIF)

S6 Fig Role of Cys 172 of Grx4 in the interaction between Grx4 and Fep1 and in the activation of Fep1-dependent genes. (A) The interaction between Fep1 and Grx4 is only partially dependent on the presence of the Fe-S cluster. Stains NG108 (*fep1-myc*), NG115 (*grx4-GFP*), NG109 (*fep1-myc grx4-GFP*) and JE11 (*fep1-myc grx4*.*C172S-GFP*) were treated or not with 0.25 mM DIP for the indicated times. Total native protein extracts were immuno-precipitated with GFP-trap beads. Immuno-precipitates were analyzed by SDS–PAGE and blotted with anti-Myc or anti-GFP antibodies. As a loading control, whole-cell extracts were loaded (WCE). (B) Upon long DIP treatments, cells expressing Grx4.C172S can promote Fep1 inactivation. Total RNA from YE cultures of strains 972 (WT), NG81 (*Δgrx4*) and NG86.C172S (*grx4*.*C172S*), before and after the indicated time in hours with DIP, were nalyzed by Northern blot with the probes indicated. *rRNA* and *act1* were used as loading controls.
(TIF)

S7 Fig Grx4 and Fra2 may have other roles than regulating Php4 and Fep1. (A and B) Cells lacking both Grx4 and Php4 still display severely compromised aerobic growth. (A) Total RNA from YE cultures of strains 972 (WT), NG81 (*Δgrx4*) and NG130 (*Δgrx4 Δphp4*) was analyzed as described in Fig. 1E with the Php4-dependent *pcl1* probe and the Fep1-dependent *fio1* probe. *rRNA* and *act1* were used as loading controls. (B) Serial dilutions from cultures of strains as in A were spotted on YE plates and grown under aerobic or anaerobic conditions. (C) The glutaredoxin Grx4 and the BolA-like protein Fra2 are more abundant that the transcriptional repressors Fep1 and Php4. 10 μg of total TCA extracts of strains JE5 (*fra2–13myc*), NG84 (*grx4-myc*), NG107 (*php4-myc*) and NG108 (*fep1-myc*), were analyzed by SDS-PAGE and Western blot with anti-Myc antibodies. Tubulin was used as a loading control.
(TIF)

S1 Text Materials and methods, Supporting Information. (DOCX)

S1 Table Strains used in this study. (DOCX)
