# Supplementary figures and images for: A Cascade of Iron-Containing Proteins Governs the Genetic Iron Starvation Response to Promote Iron Uptake and Inhibit Iron Storage in Fission Yeast
Source: PLoS Genet. 2015 Mar 25;11(3):e1005106. doi: 10.1371/journal.pgen.1005106 (PMC4373815; doi:10.1371/journal.pgen.1005106)

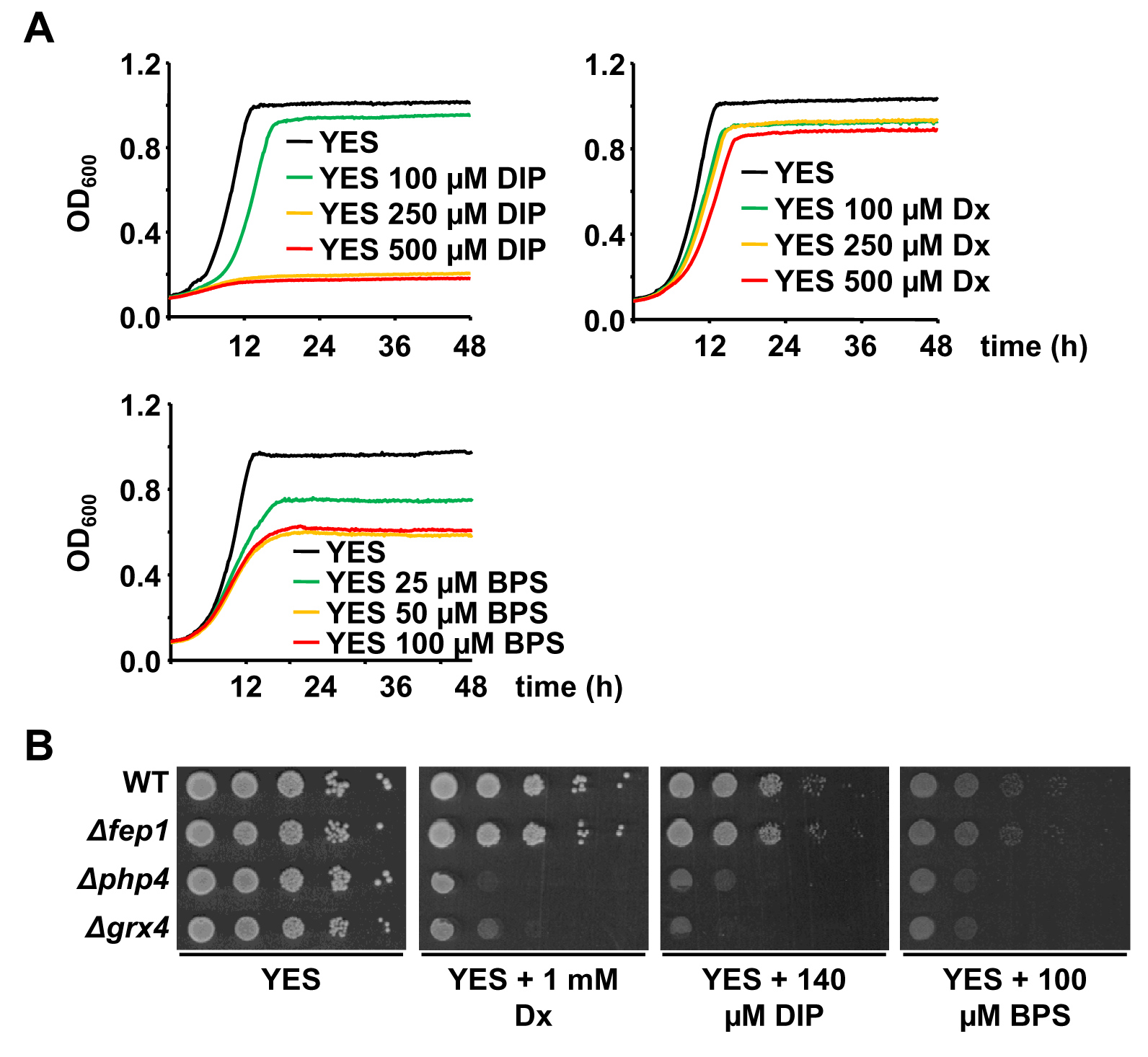

Supplement: S1 Fig — (A) Growth curves of wild-type strain in the presence or absence of the indicated concentrations of chelators. Log-phase culture (OD600: 0.1) of the wild-type strain 972 was treated or not with the indicated concentrations of DIP, Dx or BPS, and grown into microculture wells. Growth was monitored by measuring OD600 every 10 min at 30° for 48 h. (B) Cells lacking Php4 or Grx4 display growth defects in the presence of different Fe chelators. Strains 972 (WT), NG2 (Δfep1), NG40 (Δphp4) and NG81 (Δgrx4) were spotted and grown under anaerobic conditions on plates containing the indicated concentrations of Dx, DIP or BPS. (TIF) [file pgen.1005106.s001.tif]

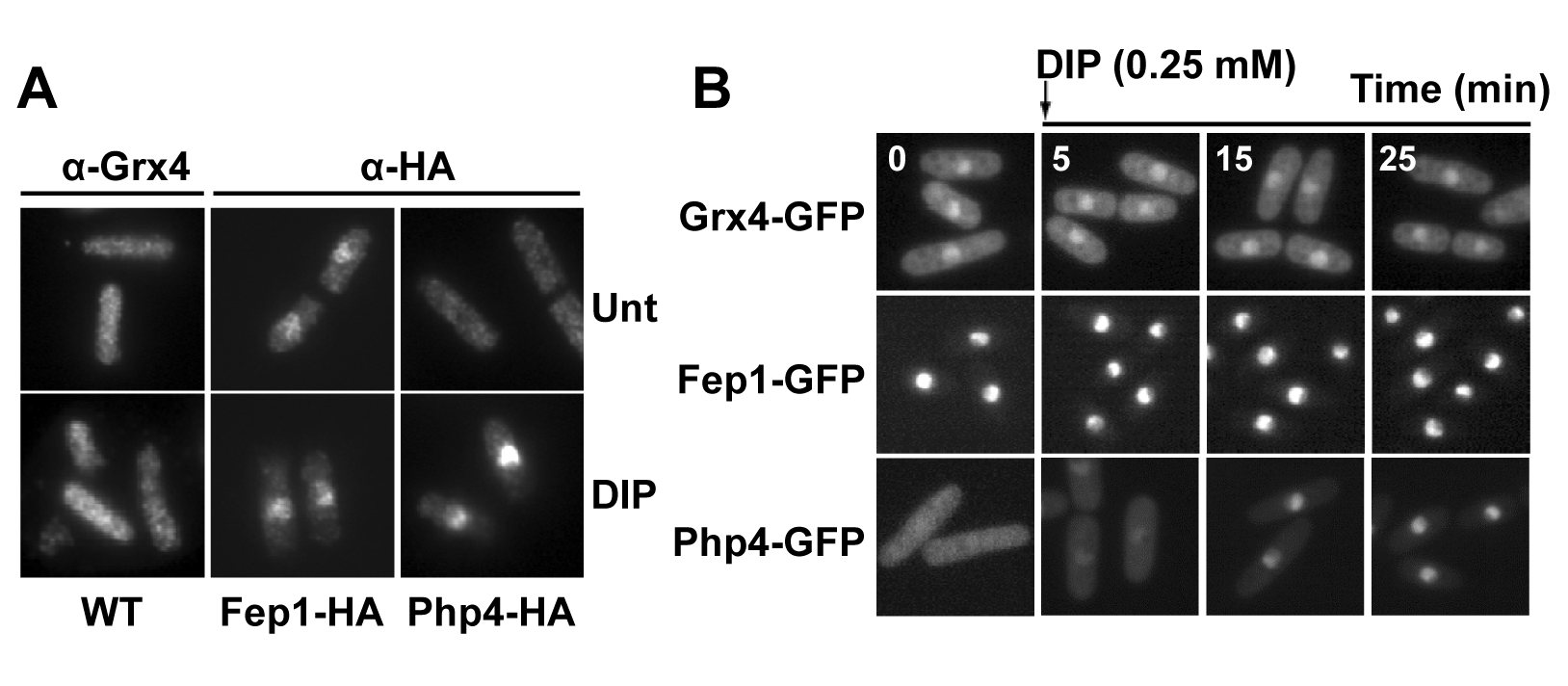

Supplement: S2 Fig — (A and B) Only Php4, but not Grx4 or Fep1, changes its sub-cellular localization upon Fe starvation. (A) Cellular localization of untagged Grx4, Php4-HA and Fep1-HA, before or after 90 min treatment with DIP, was determined by immuno-fluorescence microscopy from strains 972 (WT), NG64 (fep1-HA) and NG123 (php4-HA). (B) Cellular localization of GFP-tagged Grx4, Php4 and Fep1 was determined by fluorescence microscopy from strains NG115 (grx4-GFP), NG105 (fep1-GFP) and NG70 (php4-GFP), before and after treatment with DIP for the times indicated. (TIF) [file pgen.1005106.s002.tif]

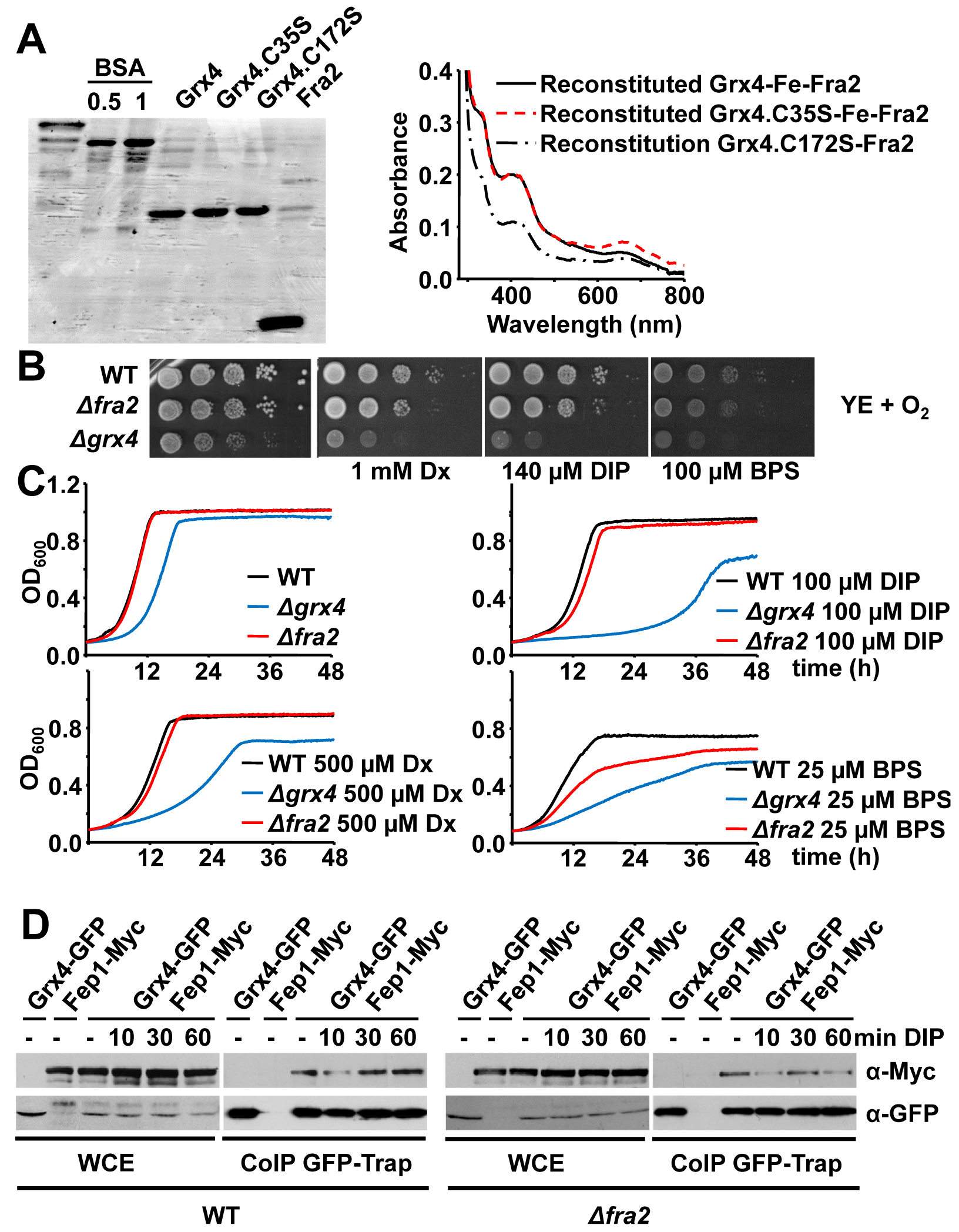

Supplement: S3 Fig — (A) Reconstitution of the Fe-S cluster of Grx4-Fra2. Electrophoretic analysis of recombinant proteins obtained in E. coli, BSA (0.5 and 1 μg) was used as a loading control. UV-visible absorption spectra of reconstituted Grx4-Fe-Fra2 (solid line), reconstituted Grx4.C35S-Fe-Fra2 (red dashed line) or reconstitution reaction with Grx4.C172S-Fra2 (black dashed line). (B) Cells lacking Fra2 display minor defects in the presence of Fe chelators such as Dx, DIP or BPS. Strains 972 (WT), NG101 (Δfra2) and NG81 (Δgrx4) were spotted and grown under aerobic conditions on YE plates containing or not the indicated concentrations of chelators. (C) Growth curves of wild-type, Δgrx4 and Δfra2 strains in the presence or absence of chelators. Growth of strains 972 (WT), NG101 (Δfra2) and NG81 (Δgrx4) was monitored as indicated in S1A Fig. (D) The in vivo interaction between Grx4 and Fep1 is not disturbed in cells lacking Fra2. Co-immunoprecipitation assays in extracts from strains NG115 (WT grx4-GFP), NG108 (WT fep1-myc), NG109 (WT grx4-GFP fep1-myc), JE6 (Δfra2 grx4-GFP), JE8 (Δfra2 fep1-myc), and JE4 (Δfra2 grx4-GFP fep1-myc) was performed as described in Fig. 1F. (TIF) [file pgen.1005106.s003.tif]

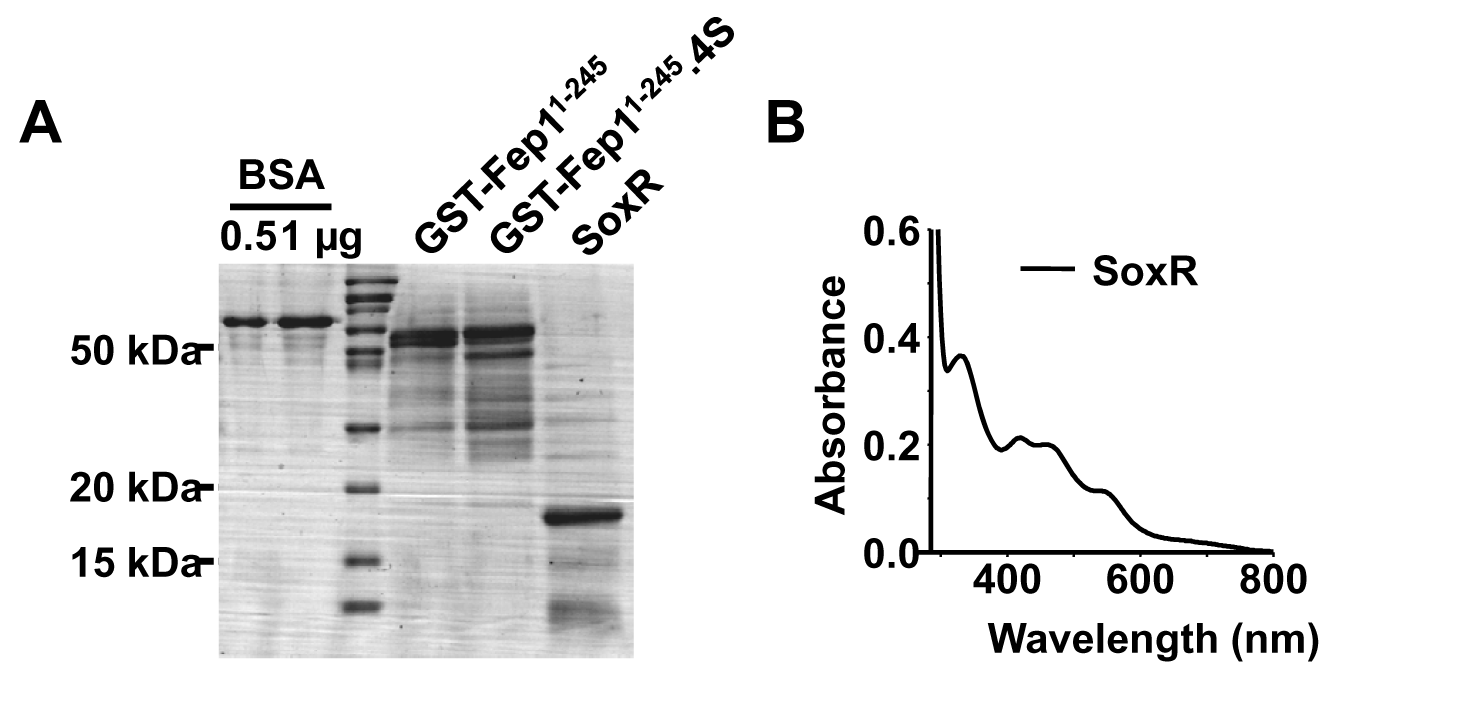

Supplement: S4 Fig — (A) Electrophoretic analysis of recombinant proteins obtained in E. coli. BSA was used as a loading control. (B) UV-Visible spectrum of purified bacterial SoxR protein. (TIF) [file pgen.1005106.s004.tif]

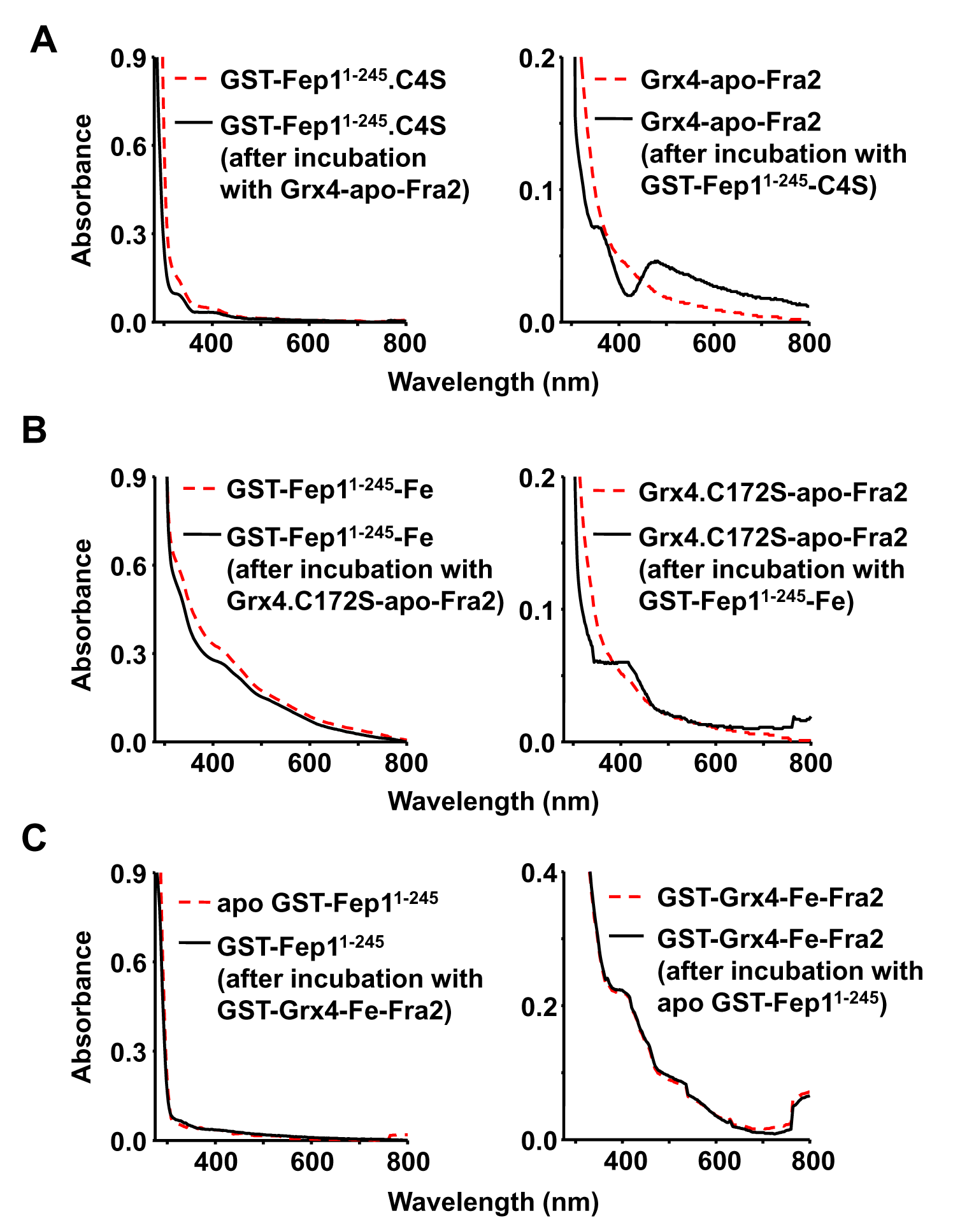

Supplement: S5 Fig — (A) UV-visible spectra of GST-tagged Fep11–245.C4S and Grx4-Fra2 were recorded before (dashed line) and after (solid line) incubation in a 1:1 protein ratio and protein separation through GSH-affinity chromatography. (B) UV-visible spectra of GST-tagged Fep11–245 and Grx4.C172S-Fra2 were recorded before (dashed line) and after (solid line) incubation in a 1:1 protein ratio and protein separation through GSH-affinity chromatography. (C) GST-Grx4-Fe-Fra2 cannot transfer Fe to apo-Fep11–245 in our in vitro system. UV/visible spectra of GST-tagged apo-Fep11–245 (left) or GST-Grx4-Fe-Fra2 (right) were recorded before (dashed line) and after (solid line) incubation in a 1:1 protein ratio and protein separation through GSH-affinity chromatography. (TIF) [file pgen.1005106.s005.tif]

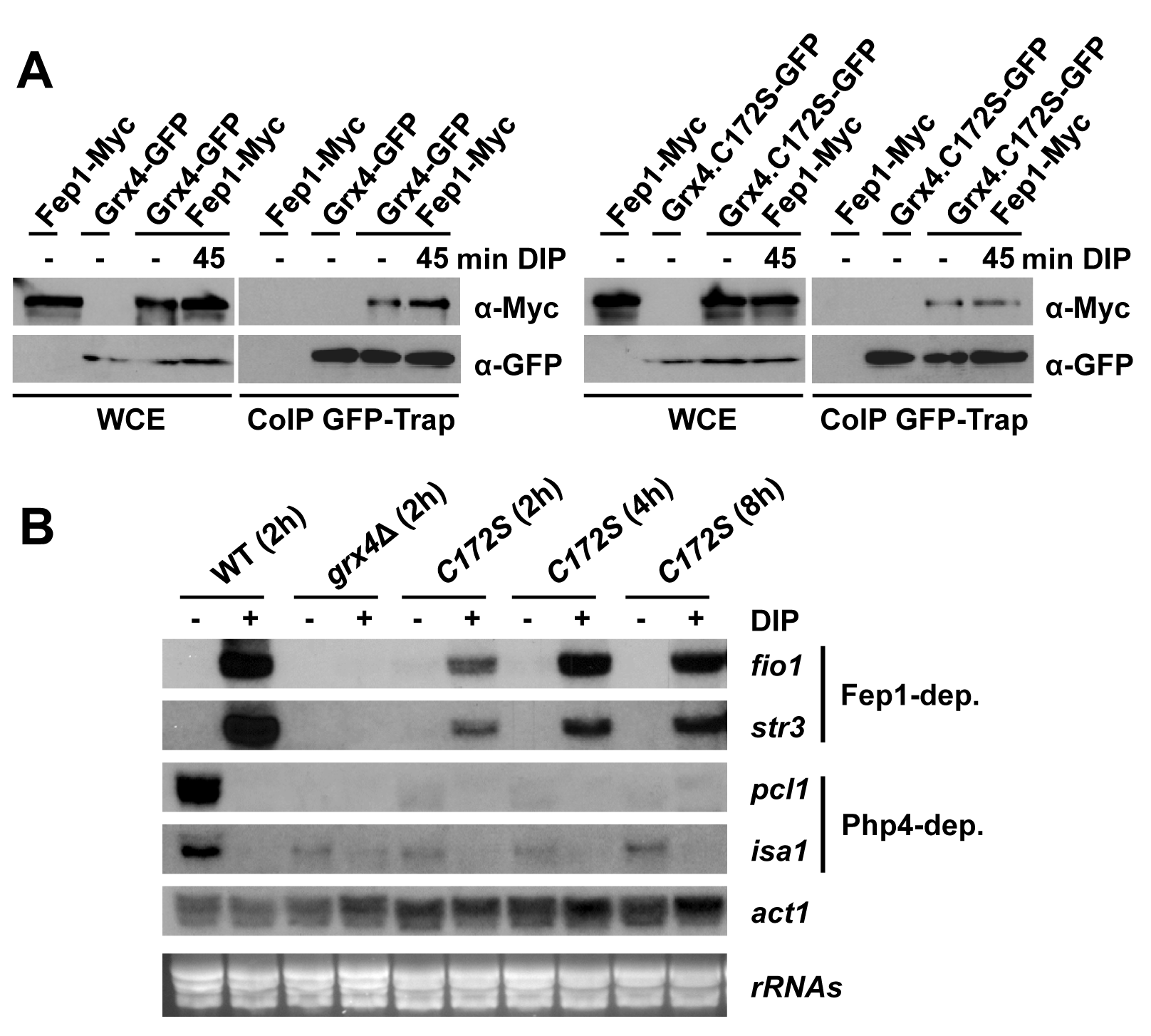

Supplement: S6 Fig — (A) The interaction between Fep1 and Grx4 is only partially dependent on the presence of the Fe-S cluster. Stains NG108 (fep1-myc), NG115 (grx4-GFP), NG109 (fep1-myc grx4-GFP) and JE11 (fep1-myc grx4.C172S-GFP) were treated or not with 0.25 mM DIP for the indicated times. Total native protein extracts were immuno-precipitated with GFP-trap beads. Immuno-precipitates were analyzed by SDS–PAGE and blotted with anti-Myc or anti-GFP antibodies. As a loading control, whole-cell extracts were loaded (WCE). (B) Upon long DIP treatments, cells expressing Grx4.C172S can promote Fep1 inactivation. Total RNA from YE cultures of strains 972 (WT), NG81 (Δgrx4) and NG86.C172S (grx4.C172S), before and after the indicated time in hours with DIP, were nalyzed by Northern blot with the probes indicated. rRNA and act1 were used as loading controls. (TIF) [file pgen.1005106.s006.tif]

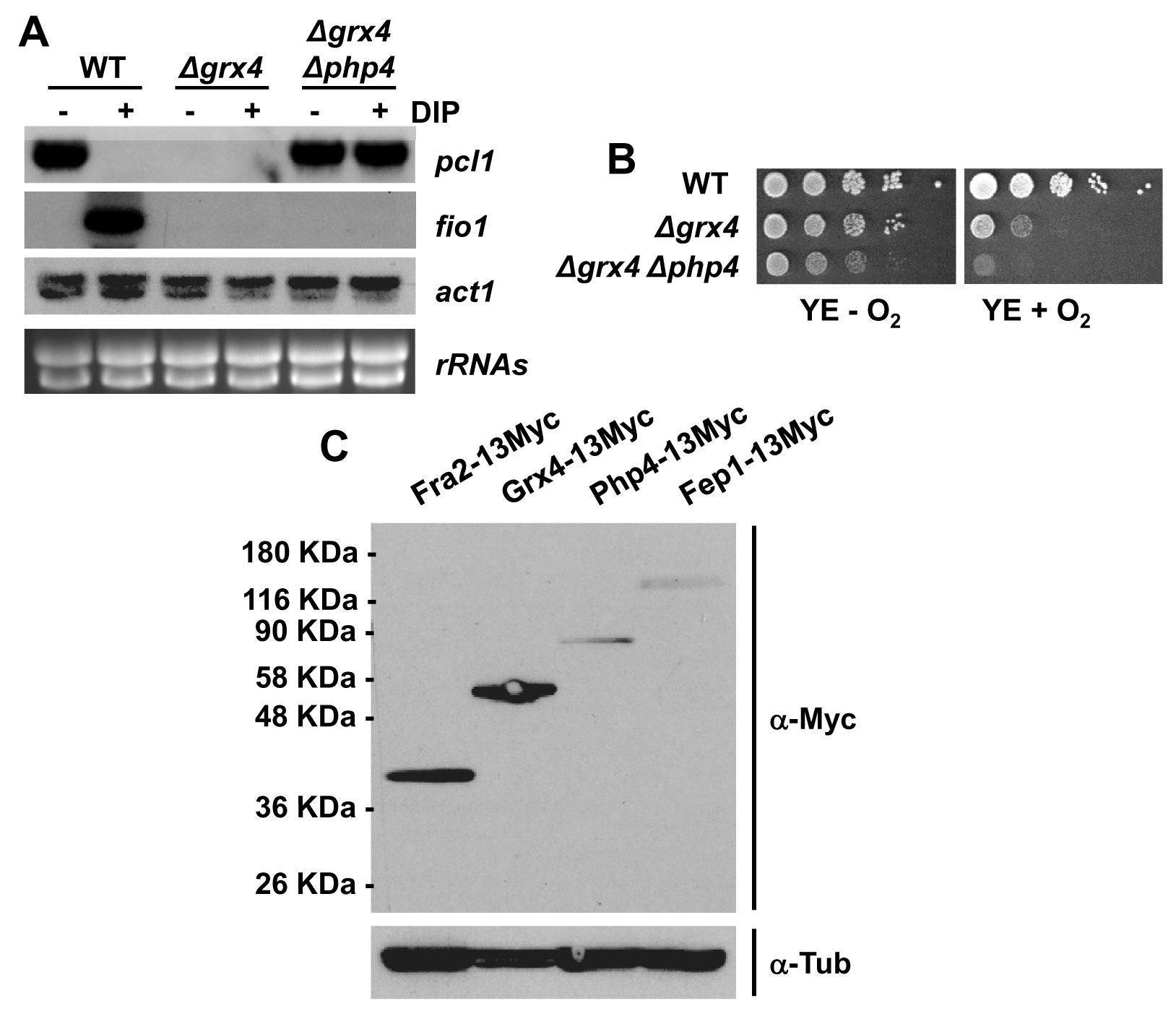

Supplement: S7 Fig — (A and B) Cells lacking both Grx4 and Php4 still display severely compromised aerobic growth. (A) Total RNA from YE cultures of strains 972 (WT), NG81 (Δgrx4) and NG130 (Δgrx4 Δphp4) was analyzed as described in Fig. 1E with the Php4-dependent pcl1 probe and the Fep1-dependent fio1 probe. rRNA and act1 were used as loading controls. (B) Serial dilutions from cultures of strains as in A were spotted on YE plates and grown under aerobic or anaerobic conditions. (C) The glutaredoxin Grx4 and the BolA-like protein Fra2 are more abundant that the transcriptional repressors Fep1 and Php4. 10 μg of total TCA extracts of strains JE5 (fra2–13myc), NG84 (grx4-myc), NG107 (php4-myc) and NG108 (fep1-myc), were analyzed by SDS-PAGE and Western blot with anti-Myc antibodies. Tubulin was used as a loading control. (TIF) [file pgen.1005106.s007.tif]
