# Supplementary material for: A Cascade of Iron-Containing Proteins Governs the Genetic Iron Starvation Response to Promote Iron Uptake and Inhibit Iron Storage in Fission Yeast
Source: PLoS Genet. 2015 Mar 25;11(3):e1005106. doi: 10.1371/journal.pgen.1005106 (PMC4373815; doi:10.1371/journal.pgen.1005106)
Supplement: S1 Text — (DOCX) [file pgen.1005106.s008.docx]

**S1 TEXT - MATERIALS AND METHODS, SUPPORTING INFORMATION**

Encinar del Dedo et al., 2015

**Strains and growth conditions**

We have constructed full deletion of the *grx4* gene and inserted the Cys mutations at the *grx4* locus following standard PCR-based methods. With the exception of HA-Fep1, expressed from a plasmid, all the strains shown in this report express tagged proteins from their own promoters and their own loci, and all of them displayed a wild-type transcriptional response to Fe starvation (data not shown). *S. pombe c*ells were grown in rich medium (YE) or synthetic minimal medium (MM) as described previously [[40](#_ENREF_1)].

**Solid sensitivity assay**

For survival on solid plates, *S. pombe* strains were grown in YE, diluted and spotted in YE or MM medium agar plates as described previously [[32](#_ENREF_2)]. The spots were allowed to dry, and the plates were incubated at 30ºC during 2 to 3 days under aerobic or anaerobic conditions (Forma Anaerobic, System Thermo electron corporation), and in the presence or not of 1 mM Fe chelator deferroxamine mesylate salt (Sigma-Aldrich), 140 µM mM DIP (Fluka) or 100 µM BPS (Sigma-Aldrich) or 4 mM ammonium ferrous sulfate (Sigma-Aldrich).

**Growth Curves**

Yeast cells were grown in YE from an initial OD_600_ of 0.1, using an assay based on automatic measurements of optical densities, as previously described [[32](#_ENREF_2)].

**RNA analysis**

Total RNA from exponentially growing *S. pombe* cells in YE, with or without treatment with the Fe chelators DIP (0.1 or 0.25 mM) or BPS (25 µM), was extracted, processed and transferred to a membrane as previously reported [[33](#_ENREF_3)]. Membranes were hybridized with [α-32P] dCTP-labeled *ﬁo1, str3, sib2, php4, pcl1, isa1* and *fep1* probes. Ribosomal *rRNAs* and/or *act1* were used as loading controls. For the experiment with the GSH auxotrophic *Δgcs1* cells, the indicated strains were grown at 30ºC in YE, then were washed three times in MM media, resuspended in MM media, and DIP was added or not for 90 min; in the case of strain *Δgcs1,* growth proceeded at 30ºC during 8 hours after the YE-to-MM shift under anaerobic conditions, prior to the addition or not of DIP for 90 min. Total RNA from cultures at an OD_600_ of 0.5 in all cases was extracted as previously described.

**Immuno-ﬂuorescence assay**

Ten ml of cells in MM were grown to an OD_600_ of 0.3 and fixed by adding 1-2% formaldehyde during 20 min at 25ºC in a water bath. Pelleted cells were washed twice in cold PBS. Cells were resuspended in 0.4 ml of preincubation buffer (20 mM citric acid, 20 mM Na_2_HPO4, 40 mM EDTA pH 8.0) with 30 mM of β-mercaptoethanol and were incubated at 30ºC for 10 min in a water bath. Cells were collected by centrifugation for 1 min at 8,000 rpm, and pellets were resuspended in 0.2 ml sorbitol-Tris buffer (1 M sorbitol, 50 mM Tris-HCl pH 7.4) with 10 mM β-mercaptoethanol and 2.25 mg/ml of zymolyase 20T (ICN biochemical); incubation proceeded at 30ºC for 40 min in a water bath. Cell wall digestion was confirmed by adding 1% SDS to 2 μl of cells and observing lysis at the microscope. Cells were pelleted at 6,000 rpm for 1 min, and pellets were washed three times with 1 ml of cold PEMS (100 mM PIPES, 1 mM EGTA, 1 mM MgSO4, 1.2 M sorbitol, pH 6.5-6.9). Then, cells were incubated in 1 ml of cold PEMS with 1% Triton X-100 during 1 min at room temperature to permeabilize nuclear membranes. Cells were pelleted at 6,000 rpm for 1 min, and washed three times with 1 ml of PEM (100 mM PIPES pH 6.9, 1 mM EGTA, 1 mM MgSO4). Cells were resuspended in 1 ml of PEMBAL (100 mM PIPES, 1 mM EGTA, 1 mM MgSO4, 3% BSA, 0.1% NaN3, 100 mM lysine hydrochloride) and incubated rocking during 20 min at room temperature. Cells were pelleted again and resuspended in 100 μl of PEMBAL containing 1:200 dilution of polyclonal anti-Grx4, or monoclonal anti-HA (12CA5) antibodies. Incubation proceeded rocking overnight at room temperature. Cells were pelleted, washed three times in PEMBAL, rocking during 10 min at room temperature each time. Pelleted cells were then resuspended in 100 μl of PEMBAL plus 1:500 dilution of secondary antibody [Cy2 AffiniPure Donkey anti-rabbit IgG (H+L) (ref. 711-225-152, Jackson) or Alexa Fluor 555 goat anti-mouse IgG (ref. A21424, Invitrogen)], and incubated by rocking 5-7 h at room temperature in the darkness. Cells were pelleted and washed three times in PEMBAL, rocking them during 10 min at room temperature each time. Cells were resuspended in 100 μl of PEMBAL, and analyzed directly by fluorescence microscopy as described previously [[34](#_ENREF_4)].

**Co-immunoprecipitation analysis**

Cells from 100 ml of MM cultures at an OD_600_ of 0.5 (1×10^7^ cells) were processed as described [[35](#_ENREF_5)]. Samples were separated by 10% SDS-polyacrylamide gel electrophoresis (PAGE) and detected by immuno-blotting with monoclonal anti-Myc (9E10) or polyclonal anti-GFP antisera [3[5](#_ENREF_5)].

**Plasmids**

The *grx4* coding sequence was PCR-amplified from an *S. pombe* cDNA library using primers specific for the Grx4-coding gene. Plasmid p389 (pGEX-2T-TEV-*grx4*) was obtained by digestion of pGEX-2T-TEV (that encode the GST tag followed by a TEV protease cleavage site; kindly provided by Xavi Gomis-Ruth) with *Bam*HI and *Sma*I and ligation with a PCR-amplified *grx4* open reading frame (ORF) flanked with the same sites. The plasmid p400 (pGEX-2T-TEV-*HA-grx4*) was obtained by digestion of plasmid p389 with *Bam*HI and ligation with annealed primers coding for the HA tag, flanked with *Bgl*II and *Bam*HI sites. The plasmids p400.C35S (pGEX2T-TEV-*HA-grx4.C35S*) and p400.C172S (pGEX-2T-TEV-*HA-grx4.C172S*) were generated by site directed mutagenesis using QuikChange XL (Agilent Tecnologies) following manufacturer instructions. Plasmid p443 [pGEX-2T-TEV-*HA-grx4-Shine-Dalgarno (AAGGAG)-fra2*] was obtained by digestion of p400 (pGEX-2T-TEV-*HA-grx4*) with *Sma*I and ligation with PCR-amplified *Shine-Dalgarno (AAGGAG)-fra2* *ORF* flanked with the same sites. Plasmid p514 (pGEX-2T-TEV-*fep1*) was obtained by digestion of pGEX-2T-TEV with *Bam*HI and *Eco*RI and ligation with PCR-amplified *fep1* ORF flanked with the same sites. To generate a mutated version of *fep1* with four Cys codons (Cys70, 76, 85 and 88) mutated to serine codons, commercial gene synthesis was performed (GenScript Tecnologies), and an N-terminal fragment of 380 bp (from the *Bam*HI to and internal *Hin*dIII site) of the *fep1* ORF in p514 was substituted by the mutated one to yield p514.C4S (pGEX-2T-TEV-*fep1.C4S*). The plasmids p514.NTD (pGEX-2T-TEV-*fep1^1-245^*) and p514.NTD.C4S (pGEX-2T-TEV-*fep1^1-245^.C4S*) were generated by PCR-amplification of the first 750 bp of the *fep1* ORF from plasmids p514 and p514.C4S and cloning into *Bam*HI-*Eco*RI-digested pGEX-2T-TEV. Regarding *S. pombe* expression plasmids, a DNA fragment coding for the HA epitope, flanked by *Sal*I and *Bam*HI sites, was inserted downstream of the weak *nmt81* (*n*o *m*essage in *t*hiamine) promoter of the episomal plasmid pRep.81x [[41](#_ENREF_6)]. PCR-amplified *Bam*HI-*Sma*I-flanked *fep1* and *fep1.C4S* inserts were then cloned in the HA-containing pRep.81x derivative, yielding plasmids p516.81x (pnmt81-*HA-fep1*) and p516.81x.C4S (pnmt81-*HA-fep1.C4S*)*.* Plasmid allowing the expression of His tagged IscS from *E. coli* was a gift from Dr. C.H. Lillig; the protein was expressed and purified as described [[20](#_ENREF_7)]. Plasmid pKOXR was used to over-express the *E. coli* protein SoxR, containing an [2Fe-2S] cluster, in strain XA90, as previously reported [[25](#_ENREF_8)].

**Bacteria growth conditions, analysis of the color of cell pellets, and purification of recombinant proteins**

Bacteria strain FB810 [[36](#_ENREF_9)] transformed with the pGEX-2T-TEV derivatives were inoculated into LB Broth Base with 100 µg/ml ampicilin and incubated at 37ºC for 16 hours with vigorous shaking. The overnight cultures were diluted 80-fold in 400 ml of fresh medium and incubated at 37ºC until the culture reached an optical density OD_600_ of 0.6. Isopropyl-β-thio-D-galactoside (IPTG, Sigma-Aldrich) was then added to a ﬁnal concentration of 1 mM and shaking continued at 18ºC for 18 h. To compare the color of the cell pellets due to Fe-containing protein over-expression, 3x10^8^ cells were pelleted in Elisa plates at 3000 rpm during 10 minutes, and then plates were scanned with Epson Perfection 4490 Photo Scanner. Regarding purification from IPTG-induced cultures of the GST-tagged proteins Grx4, Fra2 and Fep1, cells from 400 ml of IPTG-induced bacterial cultures were harvested, pellets were resuspended in 10 ml of STET extraction buffer (50 mM Tris-HCl, pH 8.0, 150 mM NaCl, 1 mM EDTA pH 8. 0,1% Triton X-100), and cells were lysed by sonication. Debris and unbroken cells were removed by centrifugation. Supernatants containing our GST-(HA)-tagged fusion proteins were incubated with glutathione (GSH)-Sepharose 4B beads (GE Healthcare) for 1 h at 4ºC. The beads were washed three times with NET-N (20 mM Tris-HCl pH 8.0, 0.5 mM EDTA pH 8.0, 100 mM NaCl, 0.5% NP-40) and once with TEV cleavage buffer (50 mM Tris-HCl pH 8.0 and 0.5 mM EDTA pH 8.0). In the case of Grx4 and Fra2, the GST-HA tagged fusion proteins were released from the beads by elution in TEV cleavage buffer with 1 mM fresh dithiothreitol (DTT) and 10 µg/ml of TEV protease (Invitrogen, Carlsbad, CA) overnight at 4ºC. We then added 0.1 M NaCl to the purified proteins and stored them at -80ºC. In the case of GST-Fep1, tagged proteins were eluted from the GSH-beads with elution buffer containing GSH (100 mM Tris-HCl pH 8.0, 150 mM NaCl, 20 mM GSH). Relative protein concentrations were determined by electrophoretic separation on 10% denaturing polyacrylamide gels and Coomassie staining with standards of bovine serum albumin (Sigma-Aldrich). Regarding purification of the bacterial SoxR transcription factor, *E. coli* strain XA90 transformed with pKOXR was grown in LB and SoxR expression induced as described before [[25](#_ENREF_8)], with the modification that IPTG induction was performed at 18ºC. Cells froms 400 ml of IPTG-induced cultures were harvested, pellets were resuspended in 20 ml of MOPS buffer (20 mM MOPS pH 7.6) with 0.2 M KCl, and cells were lysed by sonication. Protein extracts containing untagged SoxR were applied to a 1 x 5 cm column of cellulose phosphate P11 (Whatman) equilibrated with MOPS buffer with 0.2 M KCl. The column was then extensively washed with MOPS buffer with 0.2 M KCl and with 50 ml of MOPS buffer with 0.3 M KCl. The protein was eluted with 15 ml of MOPS buffer with 0.55 M KCl. Fractions with maximum brownish color, indicative of SoxR, were then analyzed by PAGE followed by Coomassie staining, by UV-visible spectroscopy, and used for subsequent analysis.

**Fe-S cluster reconstitution assay**

Cluster reassembly was performed under anaerobic conditions in a Forma Anaerobic System (Thermo Electron Corporation) at room temperature. Recombinant GST-Fep1^1-245^, wild-type or mutant Grx4 (50-60 µM) with or without equimolar amounts of Fra2 were incubated in 50 mM sodium phosphate buffer (pH 8.0) containing 200 mM NaCl, 0.01 molar equivalents of *E. coli* IscS, 2 molar equivalents of Fe(NH_4_)_2_(SO_4_)_2_, 2.5 molar equivalents of L-Cys, 1 mM GSH , 5 mM DTT, 10 µM pyridoxal phosphate, in a final volume of 125 µl for 2 hours. Then, the mixtures were desalted using Zeba^TM^ Spin Desalting Columns (Thermo). UV-visible spectra were recorded under anaerobic conditions or after 15 minutes of exposure to oxygen using a Shimadzu UV-1700 sphectrophotometer (PharmaSpec).

**Fe transfer reaction assay**

To monitor reconstitution of Grx4-apo-Fra2 from Fe-containing GST-Fep1^1-245^, recombinant GST-tagged proteins (GST-Fep1^1-245^ or GST-Fep1^1-245^.C4S) bound to GST-beads were incubated under anaerobic conditions during 2 hours with Grx4-apo-Fra2 or Grx4.C172S-apo-Fra2, 0.01 molar equivalents of *E. coli* IscS, 2.5 molar equivalents of L-Cys, 5 mM DTT, 10 µM pyridoxal phosphate. Then, beads and supernatant were separated by centrifugation (2 min at 3,000 rpm), the supernatants (with Grx4-Fe-Fra2, Grx4-apo-Fra2 or Grx4.C172S-apo-Fra2) were loaded onto Zeba^TM^ Spin Desalting Columns (Thermo) equilibrated with 100 mM Tris-HCl pH 8.0, 150 mM NaCl. The eluted fractions were analyzed by UV-visible spectroscopy and by SDS-PAGE followed by Coomassie staining. On the other hand, the proteins (GST-Fep1^1-245^ or GST-Fep1^1-245^.C4S) bound to GSH-beads were released by incubation with elution buffer containing GSH (100 mM Tris-HCl pH 8.0, 150 mM NaCl, 20 mM GSH), and the proteins were analyzed by UV-visible spectroscopy and SDS-PAGE followed by Coomassie staining.

As a control of this reverse metal reaction, we observed no reconstitution of apo-GST-Fep1^1-245^ with Fe from GST-Grx4-Fe-Fra2. To perform this control reaction, we first prepared extracts from bacteria expressing both GST-Grx4 and Fra2 from the p443 plasmid, and then bound the GST-Grx4-Fe-Fra2 heterodimer to GSH-beads. We also obtained apo-GST-Fep1^1-245^ by purification of recombinant GST-Fep1^1-245^ with thiol-containing buffers (1 mM β-mercaptoethanol), and incubated it with equimolar concentrations of GSH-beads-bound GST-Grx4-Fe-Fra2 under anaerobic conditions during 2 hours in the presence of 0.01 molar equivalents of *E. coli* IscS, 2.5 molar equivalents of L-Cys, 5 mM DTT, 10 µM pyridoxal phosphate. Then, beads and supernatant were separated by centrifugation (2 min at 3,000 rpm), the supernatants with apo-GST-Fep1^1-245^ were loaded onto ZebaTM Spin Desalting Columns (Thermo) equilibrated with 100 mM Tris-HCl pH 8.0, 150 mM NaCl. The eluted fractions were analyzed by UV-visible spectroscopy and by SDS-PAGE followed by Coomassie staining. On the other hand, the proteins GST-Grx4-Fe-Fra2 bound to GSH-beads were released by incubation with elution buffer containing GSH (100 mM Tris-HCl pH 8.0, 150 mM NaCl, 20 mM GSH), and the proteins were analyzed by UV-visible spectroscopy and SDS-PAGE followed by Coomassie staining.

**Colorimetric assay for Fe quantiﬁcation**

Five to 40 µM recombinant GST-Fep1^1-245^, GST-Fep1^1-245^.C4S or SoxR purified samples were obtained as described above. Fe quantification was performed as described previously [[37](#_ENREF_10)]. Briefly, 250 µl of purified proteins were mixed with 250 µl of 6% HNO_3_ in screw cap tubes and incubated at 98 ºC during 16 hours. The samples were centrifuged during 5 minutes at 13,000 rpm to eliminate debris and 400 µl of the supernatant were mixed with 160 µl of sodium ascorbate (38 mg/ml, Sigma), 320 µl of ferrozine (1,7 mg/ml, Fluka) and 126 µl of ammonium acetate solution (2.5 M, Sigma), vortexed thoroughly and incubated during 1 minute at room temperature. Standard curves were prepared from 2.5-40 nmoles of FeCl_3_ dissolved in 3% nitric acid. Absorbance of Fe-chelator complex was recorded at OD_565_ in a UV-visible Ultraspec 2100-pro (Amersham Biosciences) spectrophotometer. Data was obtained from three or four independent experiments and were expressed as mean ± SEM.

**Colorimetric assay for acid labile sulfide quantification**

30 to 80 µM recombinant GST-Fep1^1-245^, GST-Fep1^1-245^.C4S or SoxR purified samples were obtained as described above. Sulfide quantification was performed as described previously [[38](#_ENREF_11)]. Briefly, 200 µl of the proteins were mixed with 600 µl of 1% zinc acetate and 50 µl of 7% NaOH. The samples were mixed and incubated for 15 minutes at room temperature. The tubes were centrifuged during 30 seconds at 3,000 rpm and 150 µl of 0,1% N,N-dimethyl-p-phenylenediamine in 5 M HCl were added at the bottom of the vial until zinc hydroxide and sulfide precipitates were dissolved. Then, 150 µl of 10 mM FeCl_3_ were added and vortexed immediately during 30 seconds. The samples were centrifuged during 10 minutes at 13,000 rpm to eliminate debris and the absorbance was measured at 670 nm. Standard curves were prepared from 5-40 nmoles of lithium sulfide. Absorbance was recorded at OD_670_ in a UV-visible Ultraspec 2100-pro (Amersham Biosciences) spectrophotometer. Data was obtained from three independent experiments and were expressed as mean ± SEM.

**SUPPLEMENTAL REFERENCES**

40. Alfa C, Fantes P, Hyams J, McLeod M, Warbrick E (1993) Experiments with Fission Yeast: A Laboratory Course Manual. Cold Spring Harbor, N.Y.: Cold Spring Harbor Laboratory.

41. Maundrell K (1993) Thiamine-repressible expression vectors pREP and pRIP for fission yeast. Gene 123: 127-130.
