# Supplementary material for: A Cascade of Iron-Containing Proteins Governs the Genetic Iron Starvation Response to Promote Iron Uptake and Inhibit Iron Storage in Fission Yeast
Source: PLoS Genet. 2015 Mar 25;11(3):e1005106. doi: 10.1371/journal.pgen.1005106 (PMC4373815; doi:10.1371/journal.pgen.1005106)
Supplement: S1 Table — (DOCX) [file pgen.1005106.s009.docx]

| **Supplemental Information, Encinar del Dedo et al. 2015**  **S1 Table. Strains used in this study** | | |
| --- | --- | --- |
| **Strain** | **Genotype** | **Origin** |
| 972 | *h^-^* | [[1](#_ENREF_1)] |
| NG40 | *h^-^ php4::kanMX6* | This work |
| NG2 | *h^-^ fep1::kanMX6* | This work |
| NG86.C35S | *h^-^ grx4.C35S* | This work |
| NG86.C172S | *h^-^ grx4.C172S* | This work |
| NG81 | *h^-^ grx4::natMX6* | This work |
| NG77 | *h^-^ gcs1::kanMX6* | This work |
| NG123 | *h^-^ php4-HA::kanMX6* | This work |
| NG64 | *h^-^ fep1-HA::kanMX6* | This work |
| NG115 | *h^+^ grx4-GFP::natMX6* | This work |
| NG105 | *h^+^ fep1-GFP::kanMX6* | This work |
| NG70 | *h^-^ php4-GFP::kanMX6 ura4-D18 leu1-32* | This work |
| NG107 | *h^+^ php4-myc::kanMX6* | This work |
| NG108 | *h^-^ fep1-myc::hygMX6* | This work |
| NG84 | *h^-^ grx4-myc::kanMX6* | This work |
| NG109 | *h^-^ fep1-myc::hygMX6 grx4-GFP::kanMX6* | This work |
| NG120 | *h^+^ grx4-GFP::natMX6 php4-myc::kanMX6* | This work |
| NG101 | *h^-^ fra2::kanMX6* | This work |
| NG130 | *h^-^ php4::kanMX6 grx4::natMX6* | This work |
| JE7 | *h^-^ gcs1::kanMX6 grx4::natMX6* | This work |
| JE9 | *h^-^ grx4.C172S-GFP::natMX6* | This work |
| JE11 | *h^-^ fep1-myc::hygMX6 grx4::grx4.C172S-GFP::natMX6* | This work |
| JE4 | *h^-^ fep1-myc::hygMX6 grx4-GFP::kanMX6 fra2::kanMX6* | This work |
| JE6 | *h^+^ grx4-GFP::kanMX6 fra2::kanMX6* | This work |
| JE8 | *h^-^ fep1-myc::hygMX6 fra2::kanMX6* | This work |
| JE3 | *h^-^ fra2-GFP::kanMX6* | This work |
| JE5 | *h^+^ fra2-myc::kanMX6* | This work |
| JE17 | *h^+^ fra2-myc::kanMX6 grx4-GFP::kanMX6* | This work |
| JE16 | *h^-^ fep1::kanMX6 leu1-32* | This work |

1. Leupold U (1970) Genetical methods for *Schizosaccharomyces pombe*. Methods Cell Physiol 4: 169-177.
